# Supplementary material for: Franckeite as a naturally occurring van der Waals heterostructure
Source: Nat Commun. 2017 Feb 13;8:14409. doi: 10.1038/ncomms14409 (PMC5316837; doi:10.1038/ncomms14409)
Supplement: Supplementary Information — Supplementary Figures, Supplementary Tables, Supplementary notes and Supplementary References [file ncomms14409-s1.pdf]

Supplementary Figures

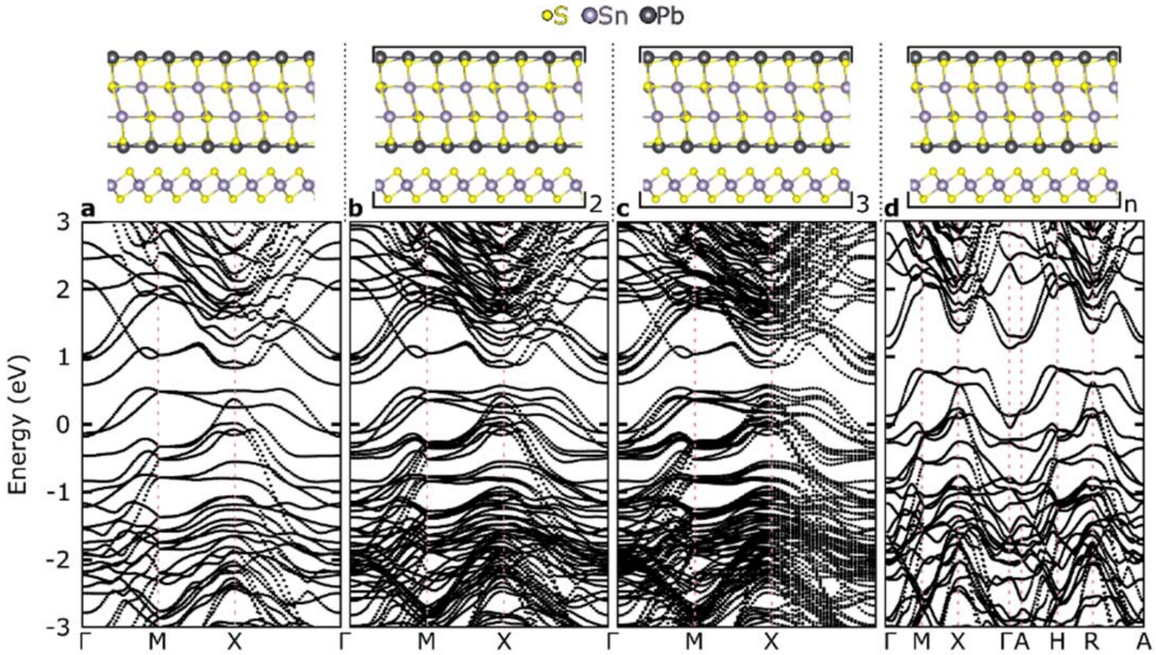

Supplementary Figure 1 | The evolution of the band structure of Franckeite with increased thickness for a n = 1. **b,** n = 2. **c,** n = 3 and **d,** bulk**.**

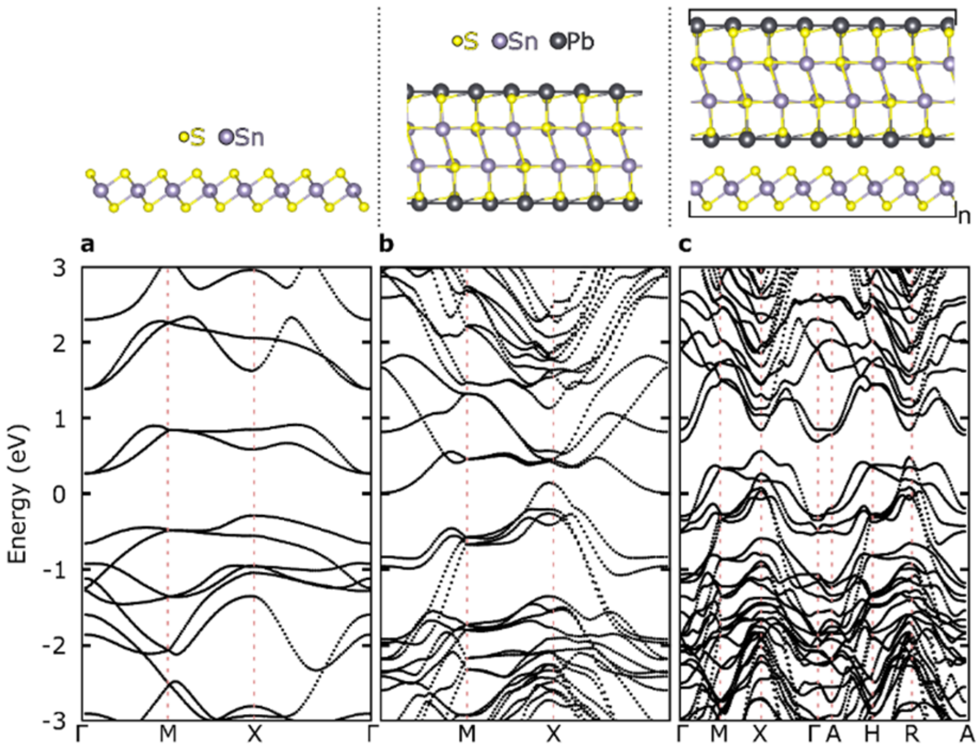

Supplementary Figure 2 | Electronic band structure of a, SnS_2 (H layer). **b**, PbSnS (Q layer) and **c**, bulk franckeite from DFT/PBE with spin-orbit coupling.

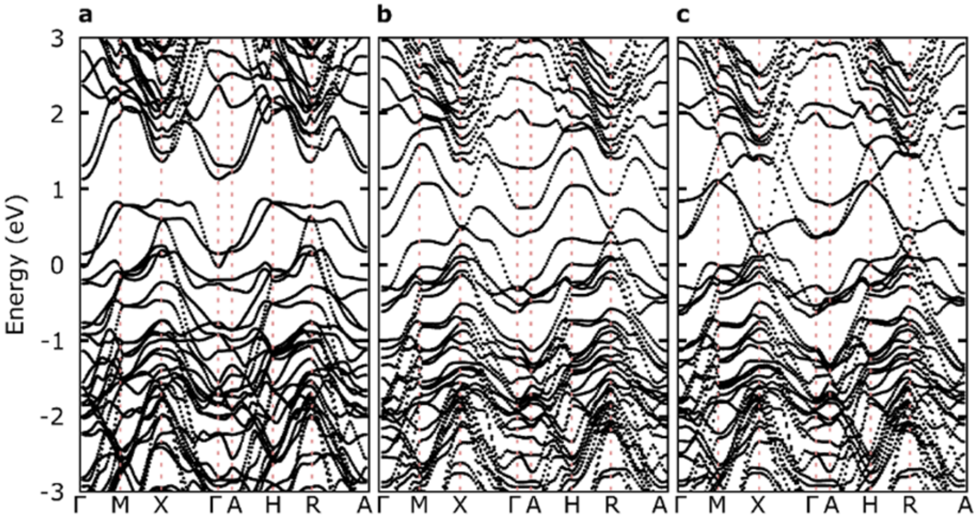

Supplementary Figure 3 | Franckeite band structure with Sb doping in the H layer. a, Calculated band structure of franckeite without Sb. **b,** Calculated band structure of franckeite when 50% of the Sn atoms have been replaced by Sb atoms in the H layer. **c,** Calculated band structure of franckeite when 100% of the Sn atoms have been replaced by Sb atoms in the H layer.

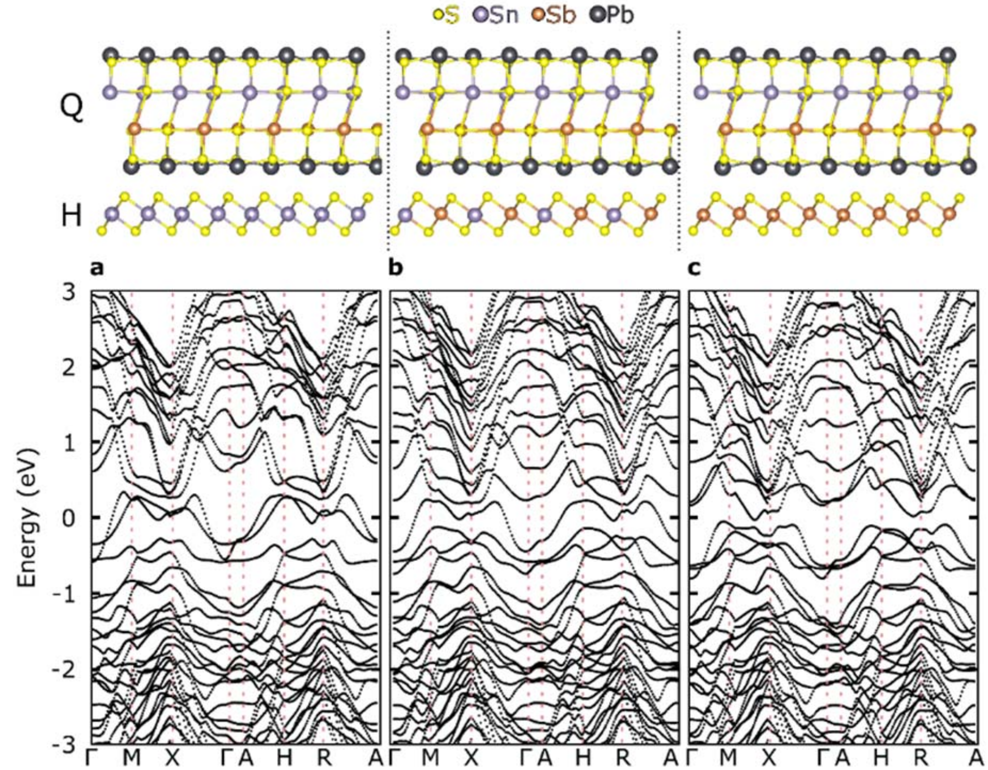

Supplementary Figure 4 | Franckeite band structure with Sb doping in the H and Q layers. a, Calculated band structure of franckeite with 50% Sn and 50% Sb in the Q layer and 0% Sb in the H layer. **b,** Calculated band structure of franckeite with 50% Sn and 50% Sb in the Q layer and 50% Sn and 50% Sb in the H layer. **c,** Calculated band structure of franckeite with 50% Sn and 50% Sb in the Q layer and 100% Sb in the H layer.

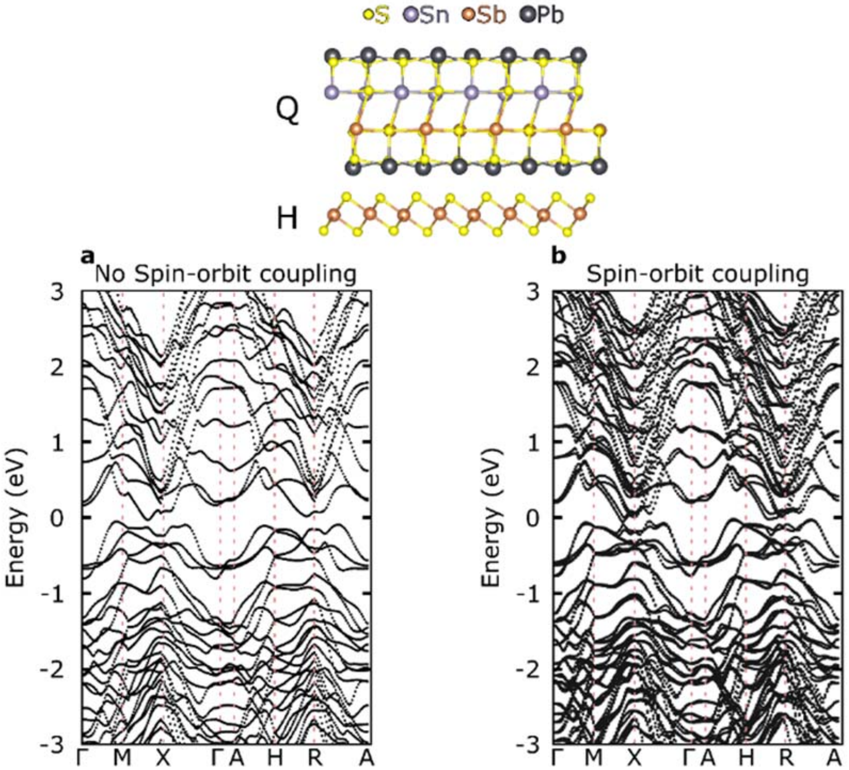

Supplementary Figure 5 | a. Calculated band structure of franckeite with 50% Sn and 50% Sb in the Q layer and 100% Sb in the H layer without considering SOC. **b,** The same but considering SOC.

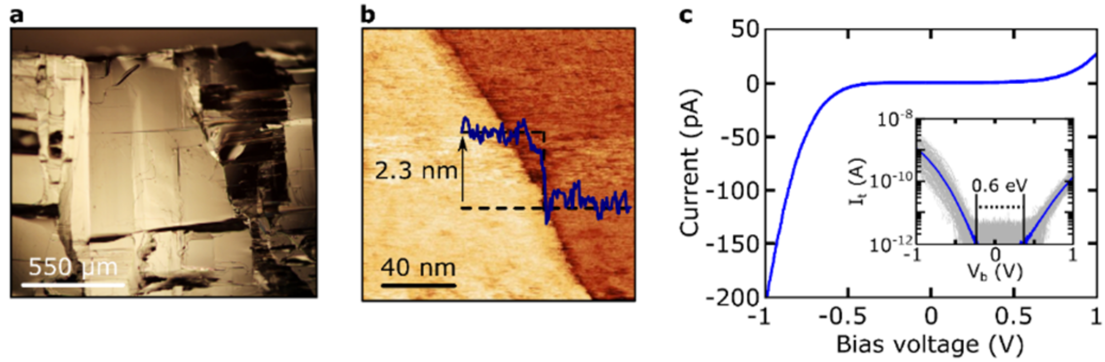

Supplementary Figure 6. a, Optical microscopy photograph of a piece of franckeite. The material presents large area flat terraces after mechanical exfoliation. **b,** STM topographic image of bulk franckeite. **c**, STS characterization of franckeite, here we show the average curve obtained from a set of 200 current-voltage curves, which clearly shows a semiconducting p-doped since the zero bias voltage (coinciding with the Fermi level) is closer to the valence band than to the conduction band. Inset: STS current-voltage curves in logarithmic scale, the valence and conduction bands values (-0.25 eV and 0.35 eV, respectively) are highlighted with arrows, yielding an electronic bandgap of ~ 0.6 eV (~0.7 eV after correction of the thermal broadening).

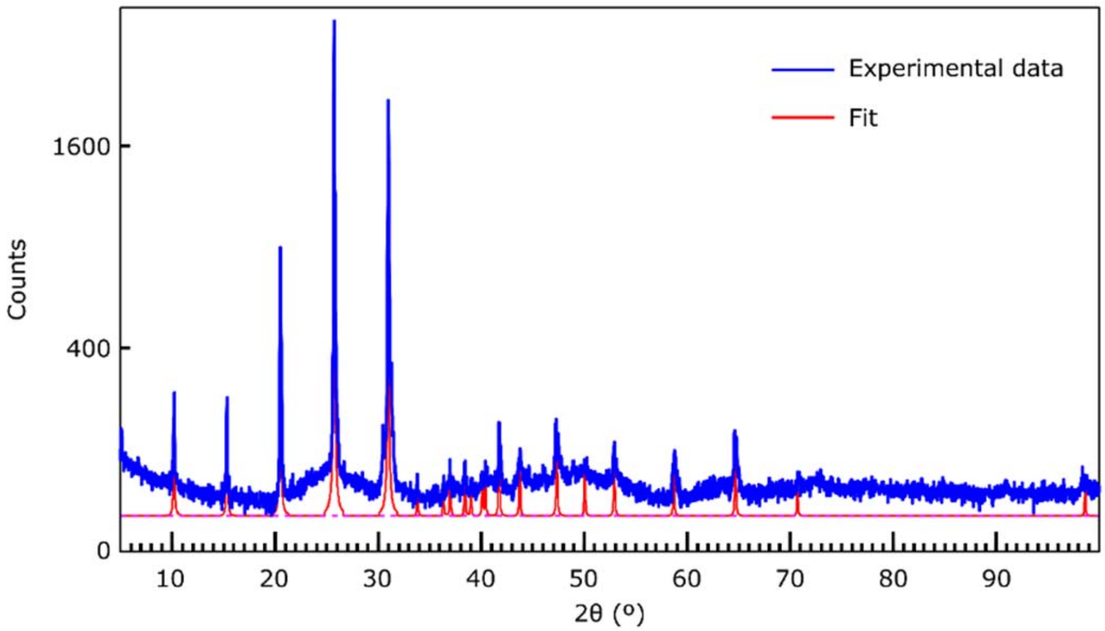

Supplementary Figure 7. X-ray diffraction pattern measured in powder franckeite.

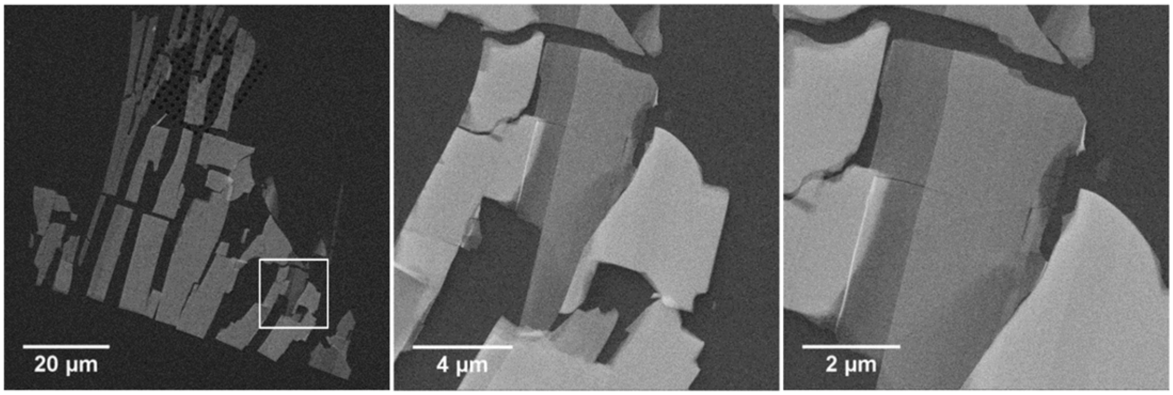

Supplementary Figure 8 | Scanning Electron Microscopy images of mechanically exfoliated franckeite. a, SEM image of several ultrathin franckeite layers deposited on a TEM grid. **b,** Zoom in on the white squared area marked in a, showing layers of various thicknesses. Conditions: 15 kV, 11 pA, working distance 6.5 mm. **c,** Zoom in on the image shown in b.

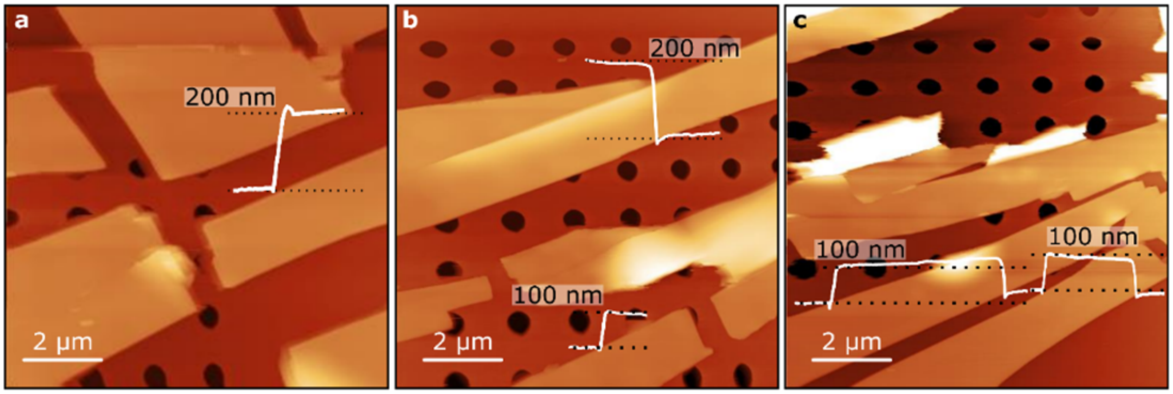

Supplementary Figure 9. a to **c**, AFM topographic images of the samples used to measured TEM in mechanically exfoliated flakes. The thickness of the different flakes ranges from 100 nm to 200 nm.

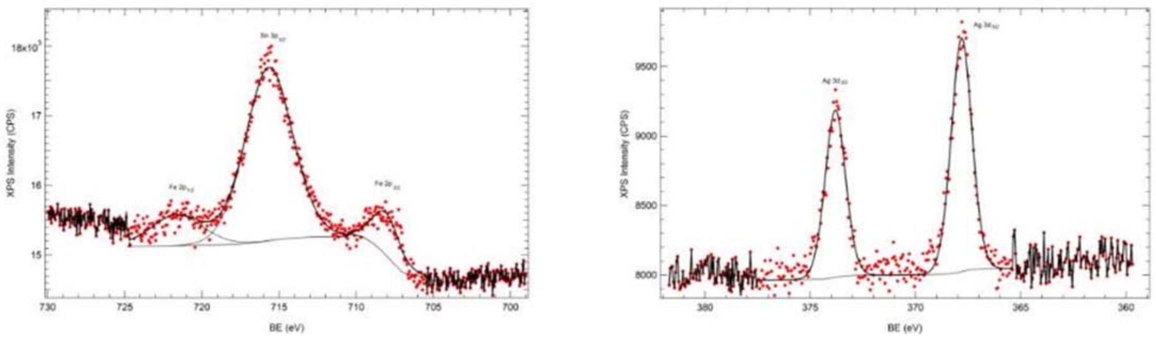

Supplementary Figure 10. XPS spectra of (a) Fe 2p region, coincident with Sn $3p_{3/2}$ core level and (b) Ag 3d region.

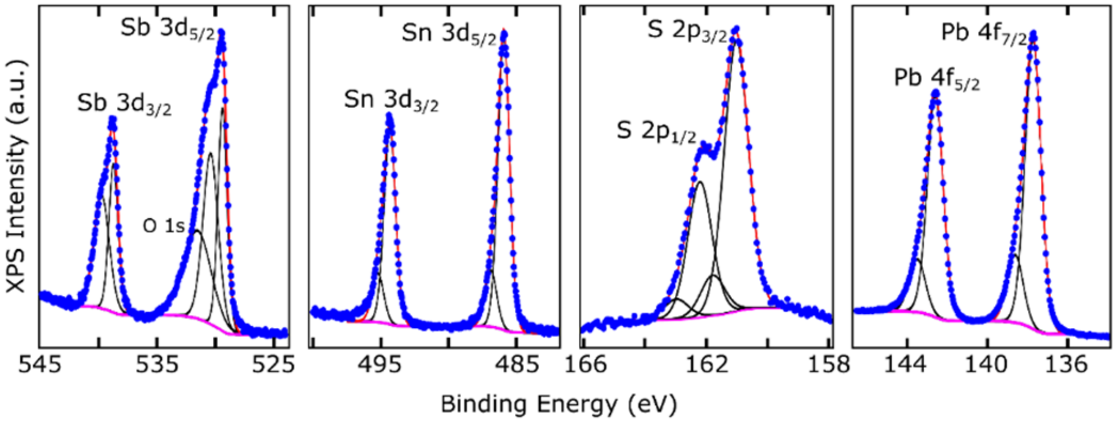

Supplementary Figure 11. XPS spectra of Sb 3d, Sn 3d, S 2p and Pb 4f regions of a franckeite mineral chip.

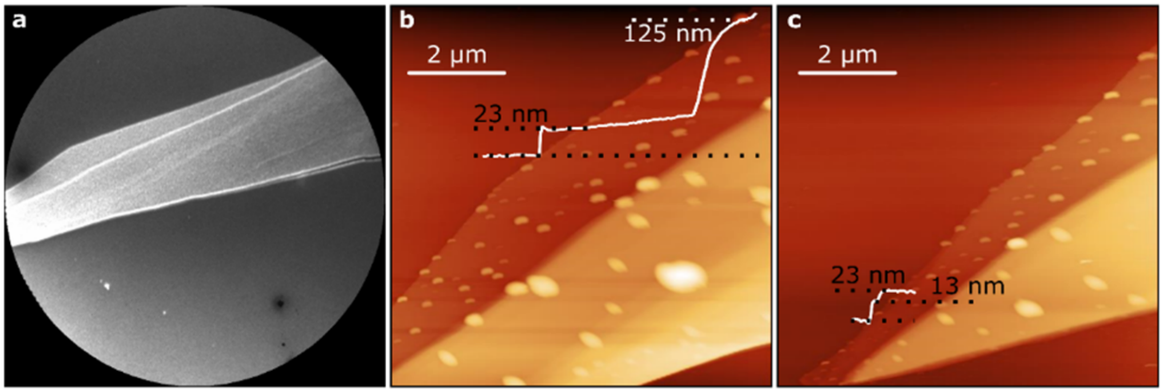

Supplementary Figure 12. a, LEEM image of the flake used to perform the micro-XPS measurements in the main text (the field of view is 50 μm and the electron energy is 0.12 eV). **b** and **c,** AFM topographic images of the flake used to perform the micro-XPS measurements in the main text.

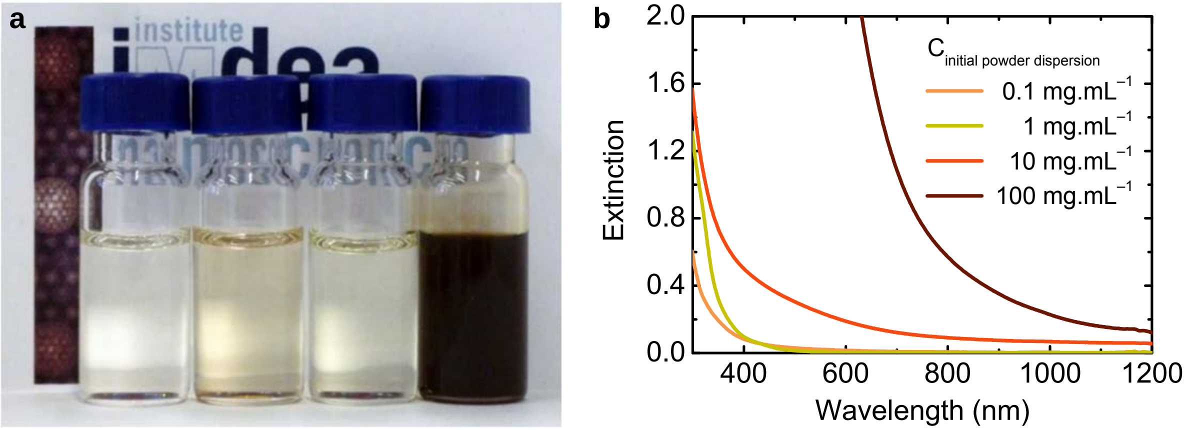

Supplementary Figure 13 | Franckeite nanosheet colloidal suspensions in NMP. a, From left to right: colloidal suspensions obtained after sonication of 0.1, 1, 10 and 100 mg·mL^{-1} franckeite powder dispersions in NMP and centrifugation. **b**, Extinction spectra of the colloidal suspensions shown in **a**.

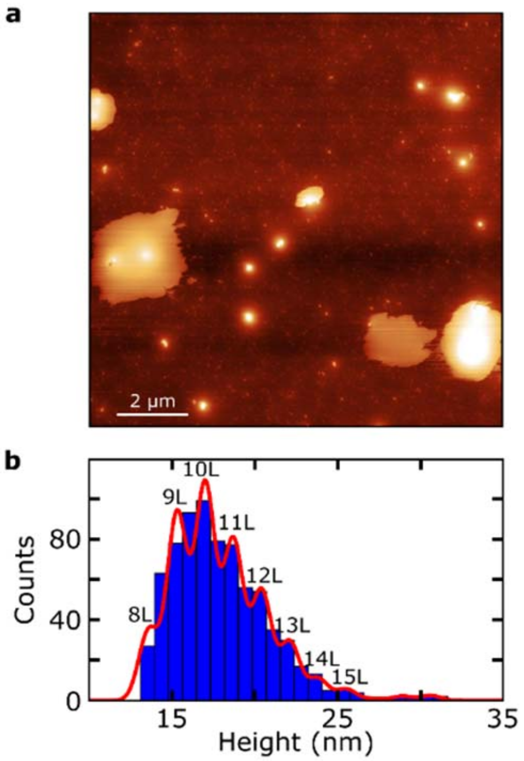

Supplementary Figure 14 | AFM of NMP-exfoliated franckeite colloidal dispersion prepared by sonication of a 100 mg·mL^{-1} dispersion. a, AFM micrograph of the sample dropcasted and dried over a freshly exfoliated mica substrate. **b**, Statistical analysis of the raw height data.

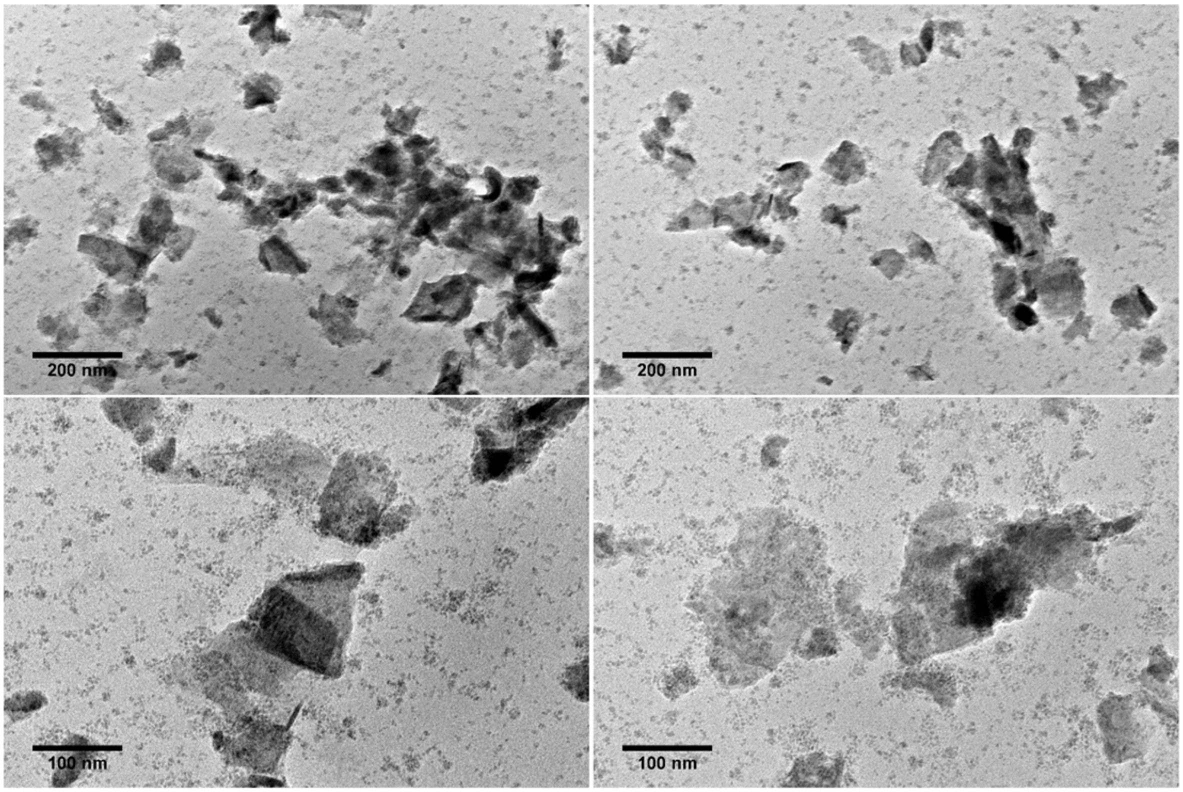

Supplementary Figure 15 | TEM images of franckeite nanosheets obtained in NMP from a 100 mg·mL^{-1} powder dispersion. Nanosheets coming from the colloidal suspension in NMP resulting from the centrifugation of the sonicated sample.

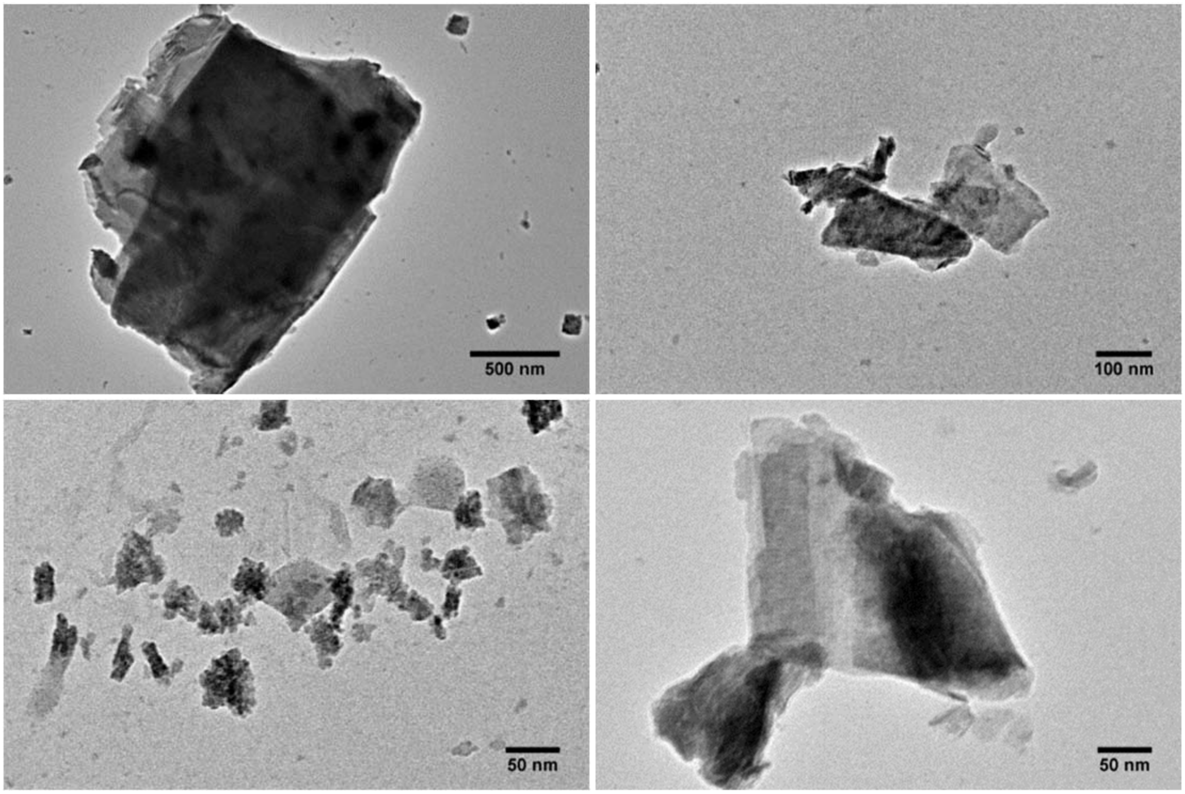

Supplementary Figure 16 | TEM images of franckeite nanosheets obtained in NMP from a 1 mg·mL^{-1} powder dispersion. Nanosheets coming from the colloidal suspension in NMP resulting from the centrifugation of the sonicated sample.

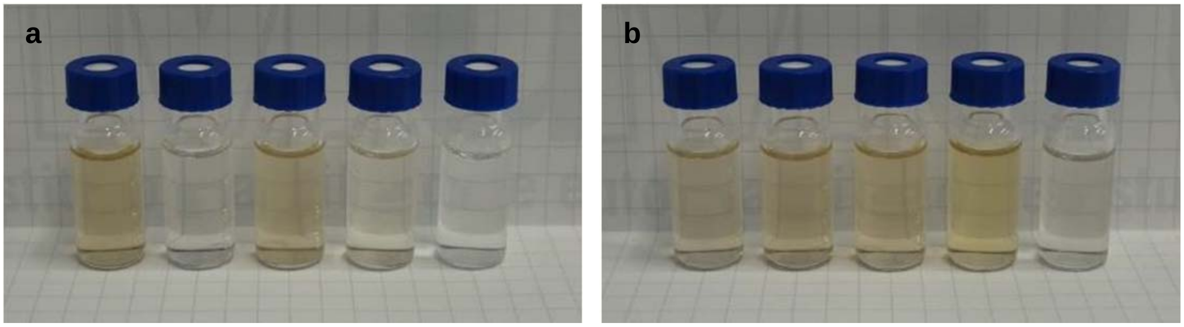

Supplementary Figure 17 | Franckeite nanosheet colloidal suspensions from exfoliation in IPA/water mixtures. a, From left to right: nanosheet suspensions obtained after sonication of 1 mg·mL^{-1} franckeite powder dispersions in IPA/water 1/0, 4/1, 1/1, 1/4 and 0/1, and subsequent centrifugation. **b,** From left to right: nanosheet suspensions obtained after IPA redispersion of the sediments from the experiments in IPA/water 1/0, 4/1, 1/1, 1/4 and 0/1, and subsequent centrifugation.

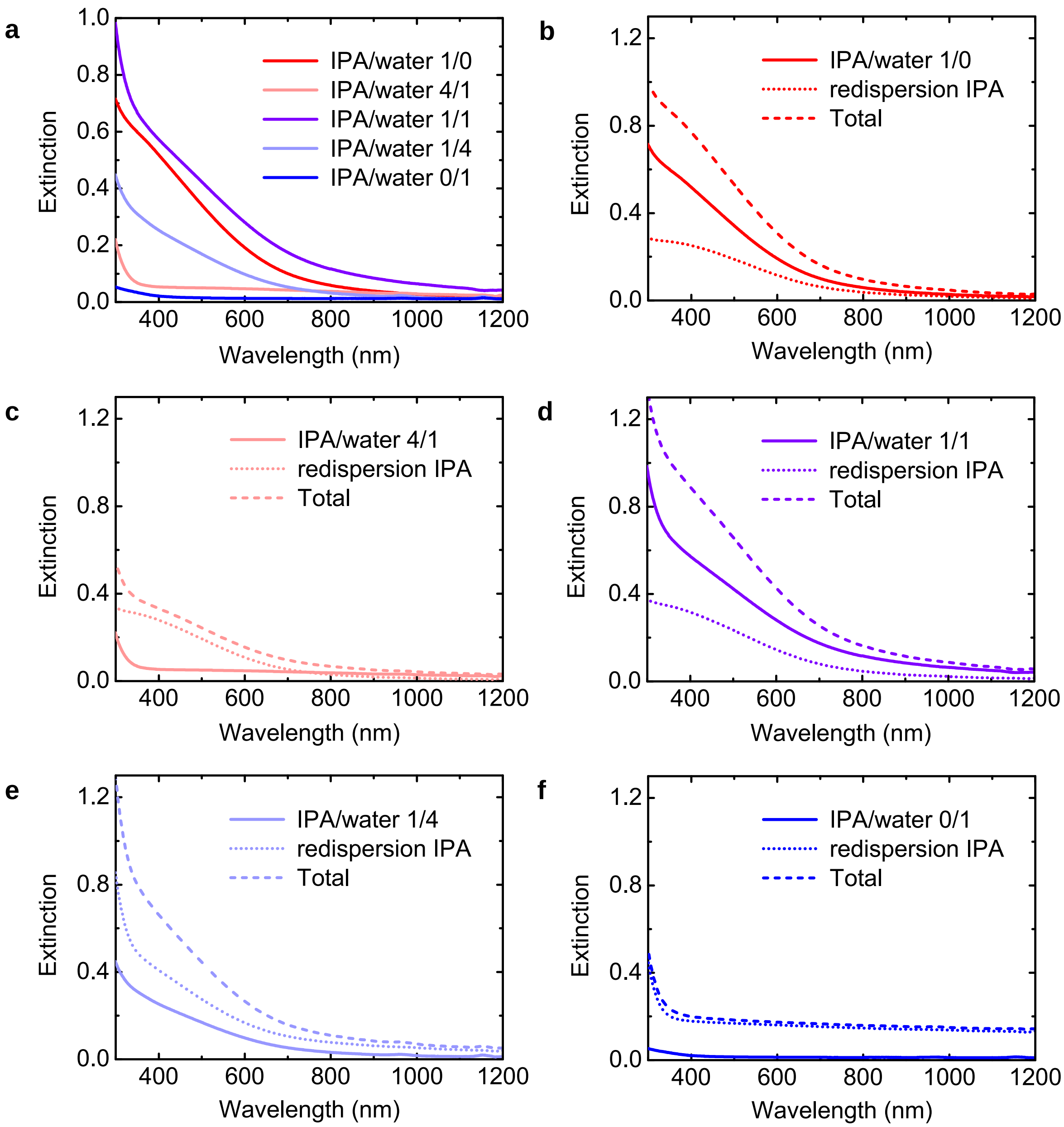

Supplementary Figure 18 | UV-Vis-NIR spectroscopy of the colloidal suspensions prepared by sonication of 1 mg·mL^{-1} franckeite dispersions in various IPA/water mixtures (v/v). a Extinction spectra of the suspensions obtained after sonication of franckeite powder in the mixture indicated (7 mL) and centrifugation. **b** Extinction spectra of the suspensions obtained after sonication of franckeite powder in the 1/0 IPA/water mixture (7 mL) and centrifugation (solid line); extinction spectra of the suspensions

obtained after redispersion of the sediment resulting from the centrifugation in the same volume of IPA (dotted line); sum of the two preceding spectra (dashed line). **c** to **f** Same as **b** for 4/1, 1/1, 1/4 and 0/1 IPA/water mixture respectively.

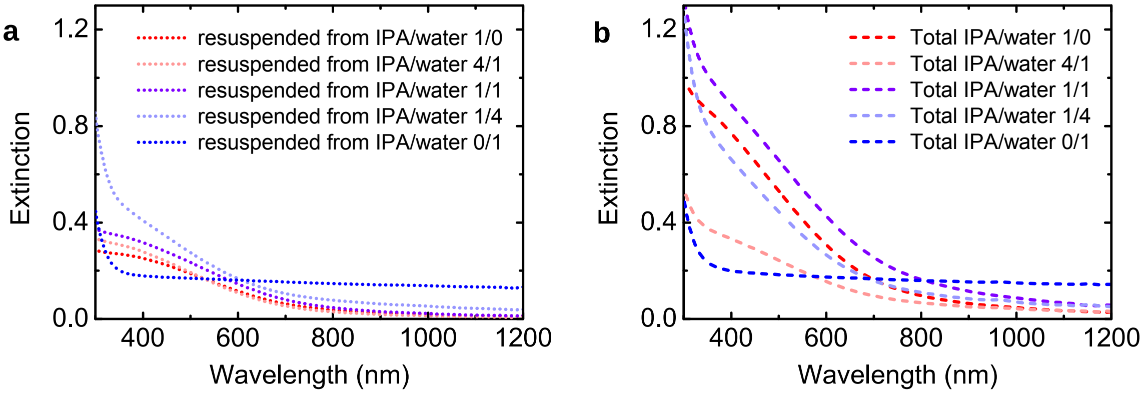

Supplementary Figure 19 | Comparison of the exfoliation processes in various IPA/water mixtures. a, Extinction spectra of recovered exfoliated franckeite in IPA from the sediment of each sample. **b,** Extinction spectra of cumulated exfoliated material from each experiment.

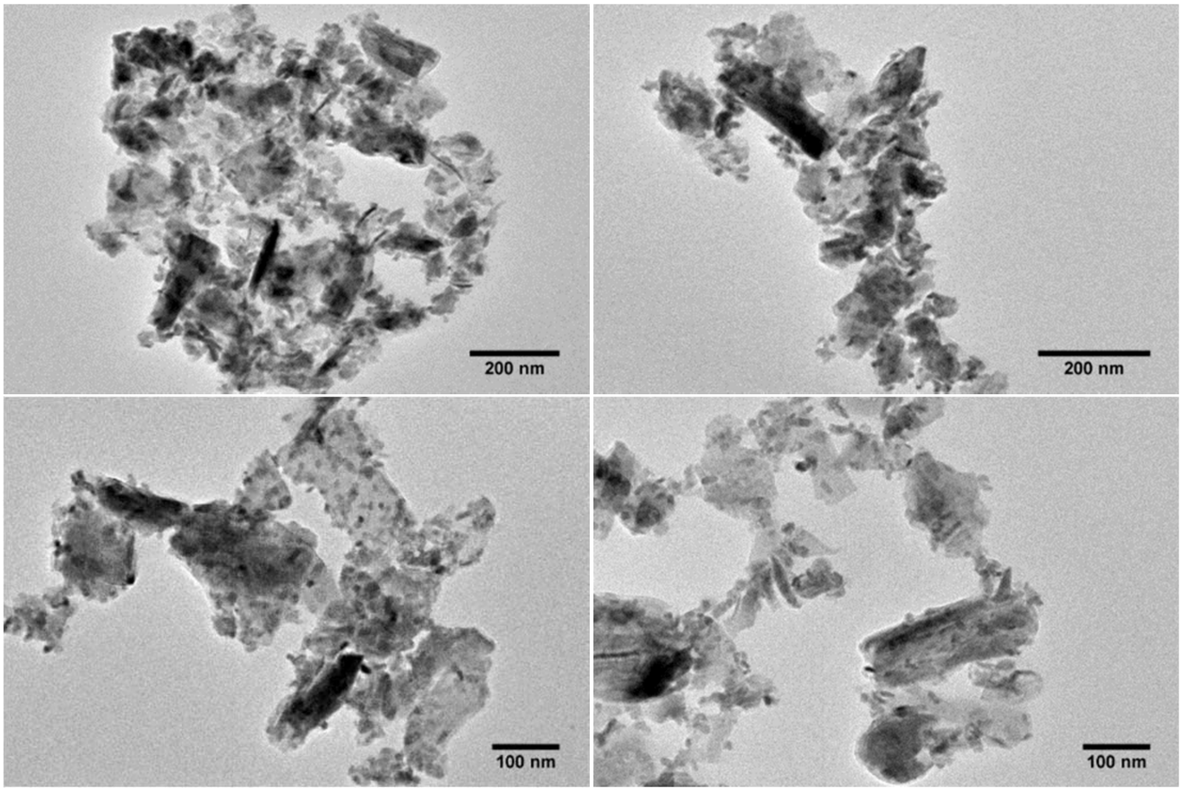

Supplementary Figure 20 | TEM images of franckeite nanosheets obtained in IPA from a 1 mg·mL^{-1} powder dispersion. Nanosheets coming from the first colloidal suspension in IPA resulting from the centrifugation of the sonicated sample.

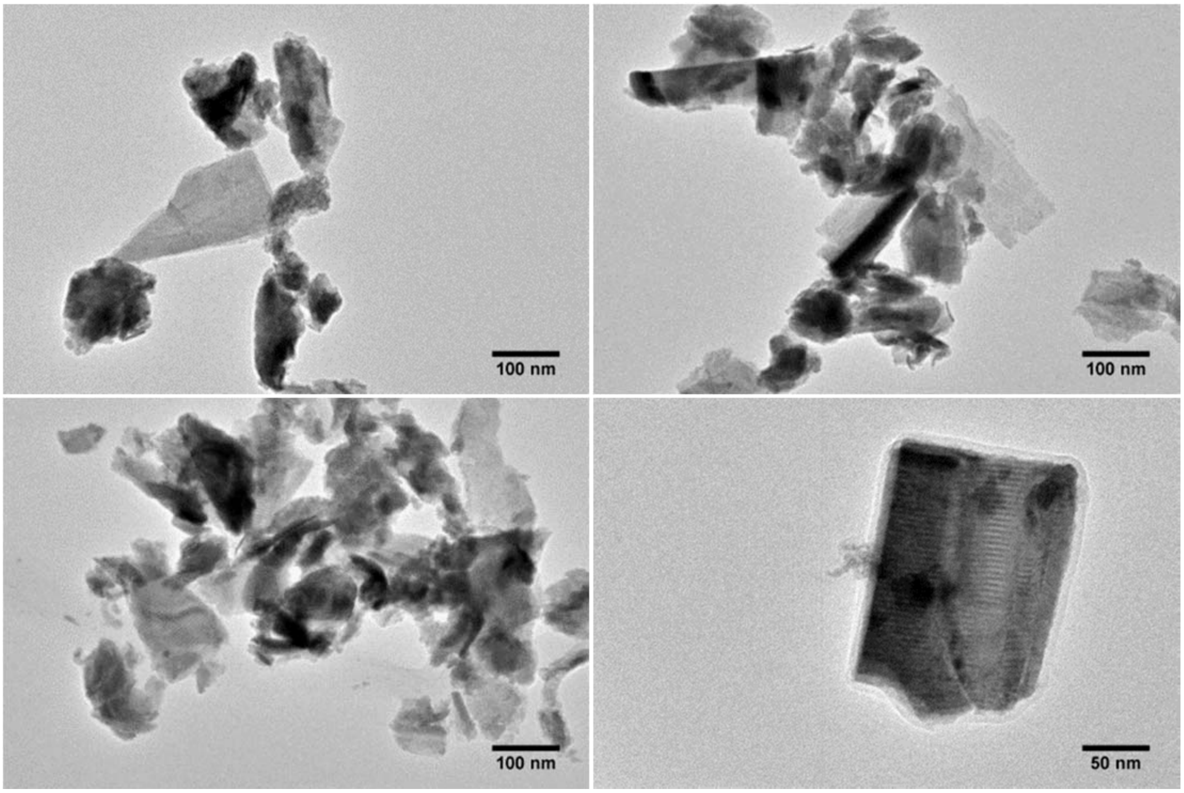

Supplementary Figure 21 | TEM images of franckeite nanosheets obtained in IPA/water 4/1 v/v from a 1 mg·mL^{-1} powder dispersion. Nanosheets recovered from the IPA redispersion of the sediment resulting from the centrifugation of the sonicated sample.

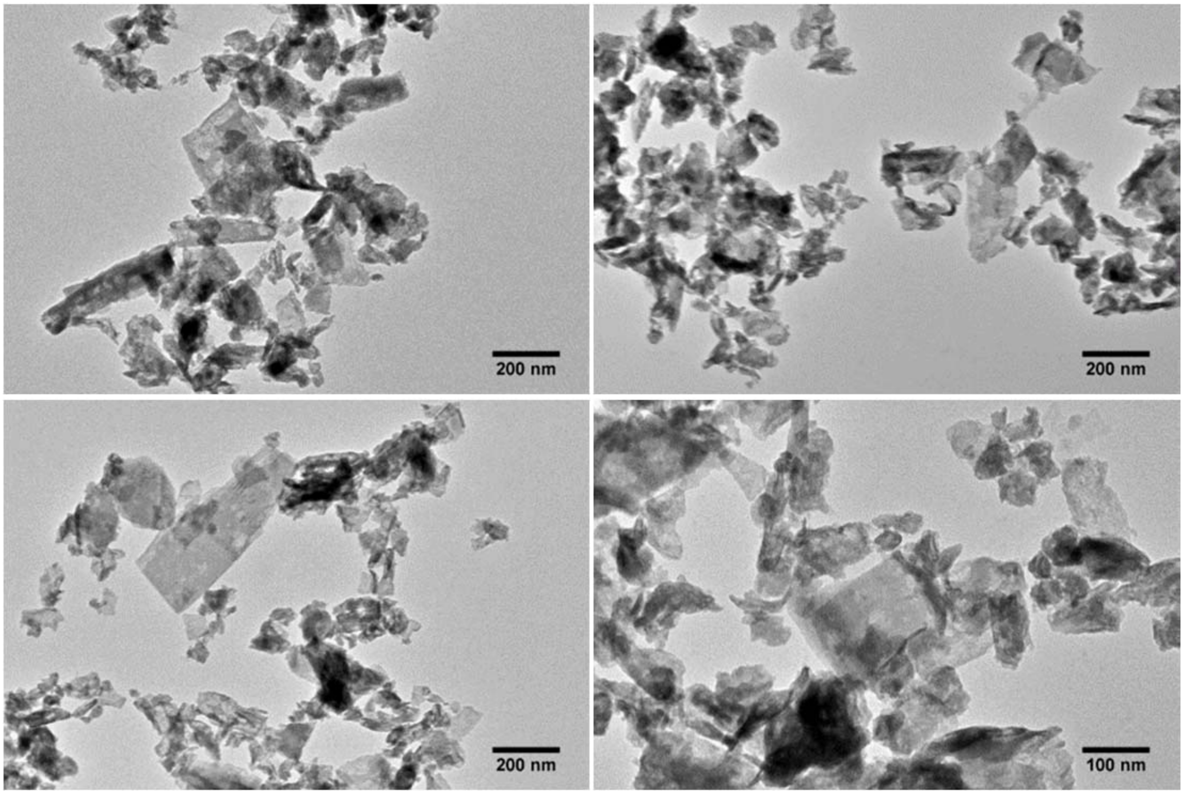

Supplementary Figure 22 | TEM images of franckeite nanosheets obtained in IPA/water 1/1 v/v from a 1 mg·mL^{-1} powder dispersion. Nanosheets coming from the colloidal suspension in IPA/water 1/1 resulting from the centrifugation of the sonicated sample.

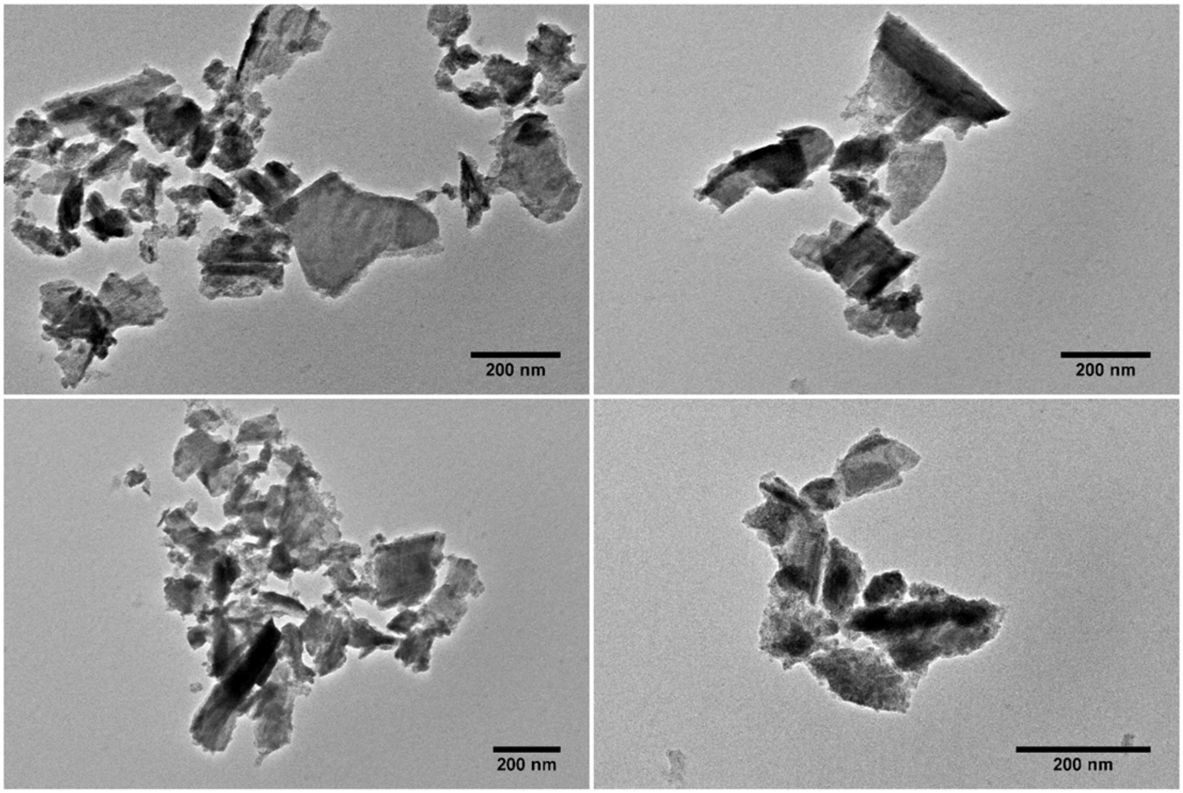

Supplementary Figure 23 | TEM images of franckeite nanosheets obtained in IPA/water 1/4 v/v from a 1 mg·mL^{-1} powder dispersion. Nanosheets coming from the colloidal suspension in IPA/water 1/4 resulting from the centrifugation of the sonicated sample.

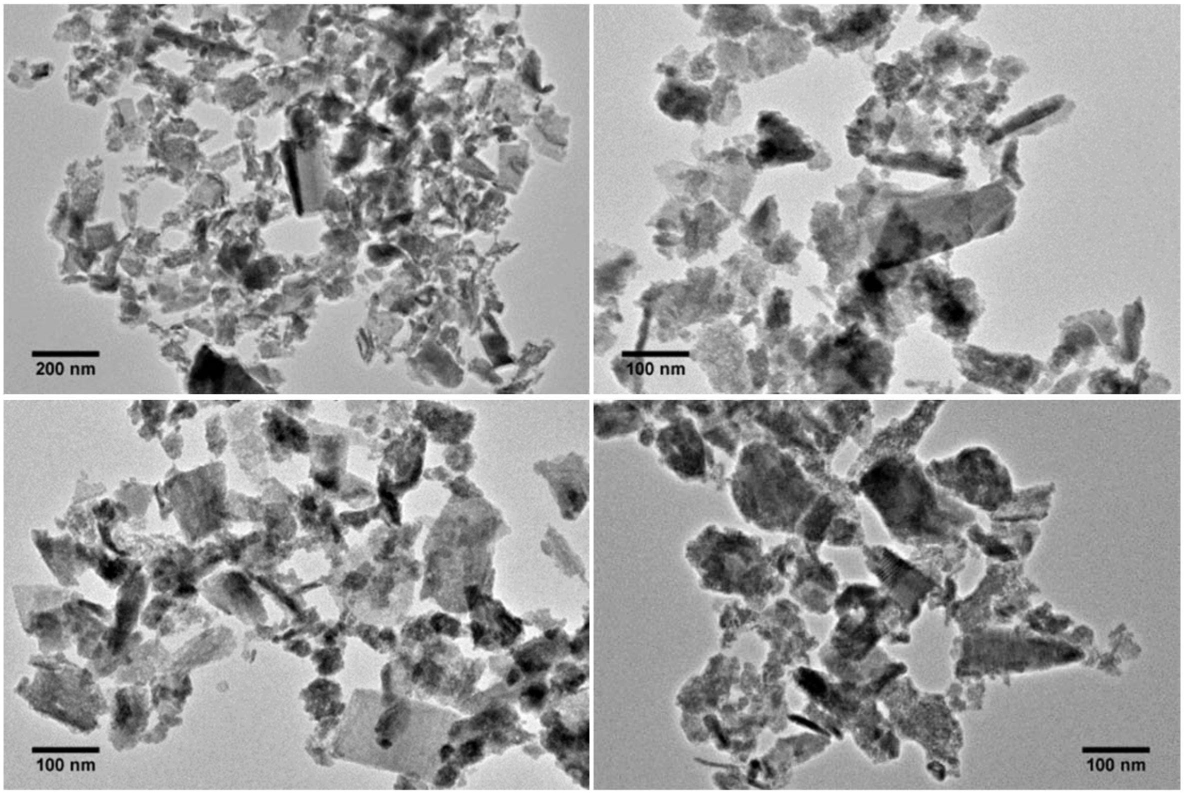

Supplementary Figure 24 | TEM images of franckeite nanosheets obtained in IPA/water 1/4 v/v from a 1 mg·mL^{-1} powder dispersion. Nanosheets recovered from the IPA redispersion of the sediment resulting from the centrifugation of the sonicated sample.

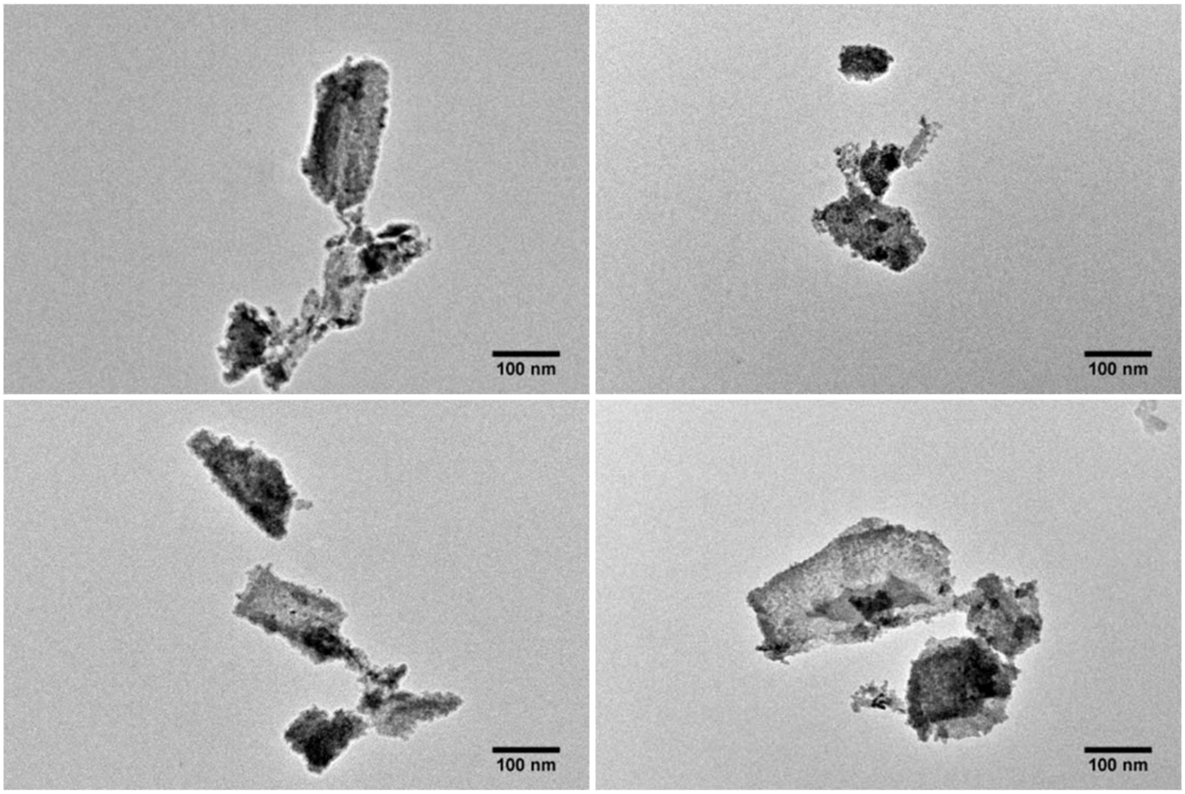

Supplementary Figure 25 | TEM images of franckeite nanosheets obtained in water from a 1 mg·mL^{-1} powder dispersion. Nanosheets recovered from the IPA redispersion of the sediment resulting from the centrifugation of the sonicated sample.

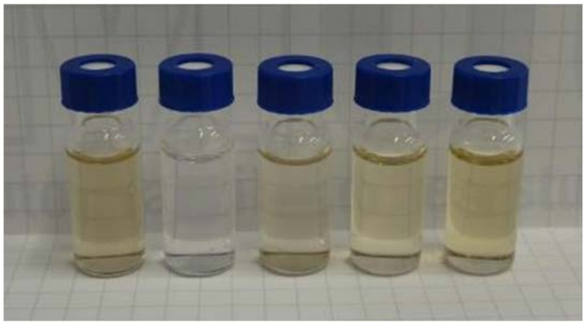

Supplementary Figure 26 | Franckeite nanosheet colloidal suspensions from exfoliation in pure polar solvents. From left to right: nanosheet suspensions obtained after sonication of 1 mg·mL^{-1} franckeite powder dispersions in IPA, water, methanol, DMF and NMP, and subsequent centrifugation.

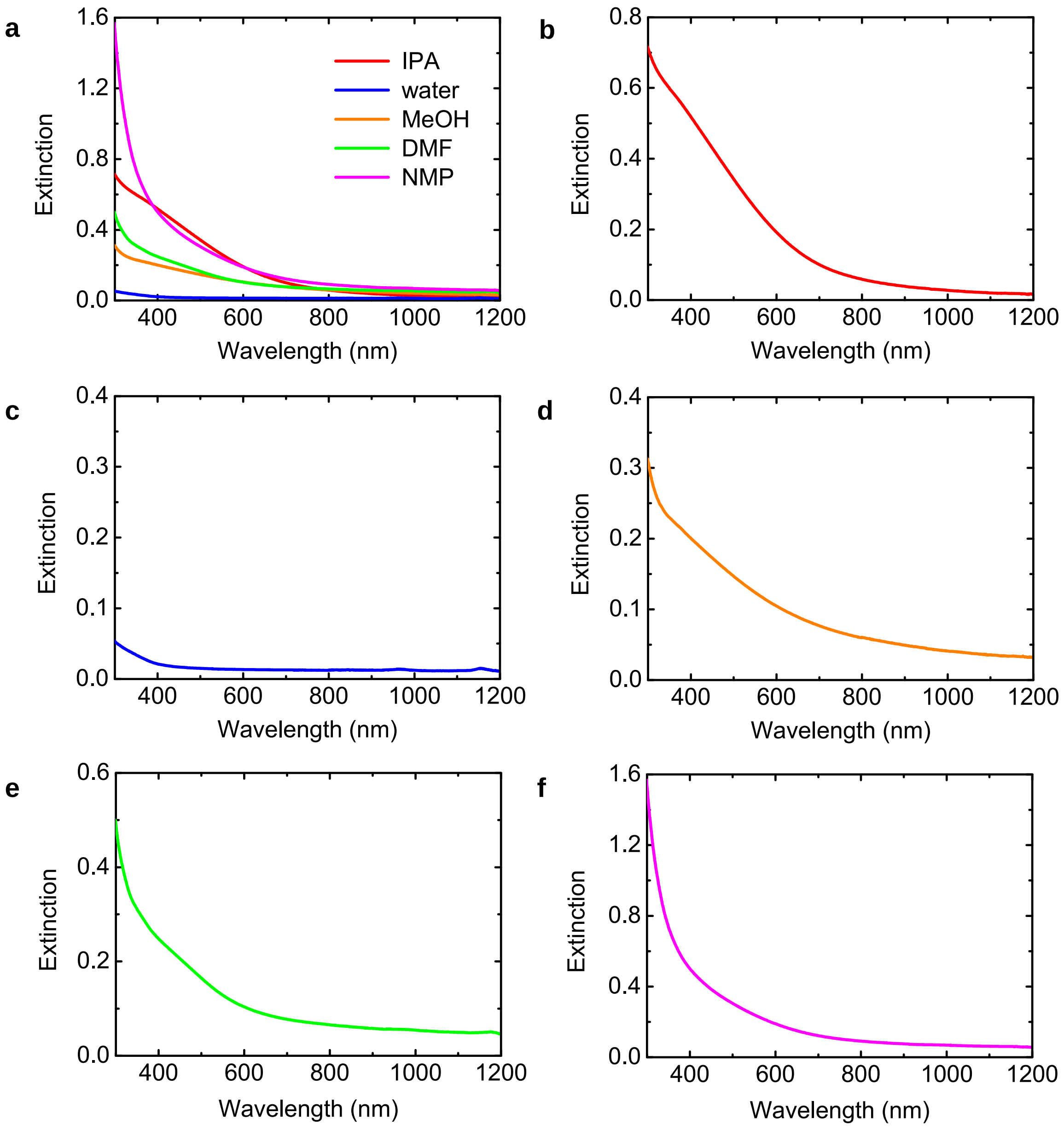

Supplementary Figure 27 | UV-Vis-NIR spectroscopy of the colloidal suspensions prepared by sonication of 1 mg·mL^{-1} franckeite dispersions in various solvents. Extinction spectra of the suspensions obtained after sonication of franckeite powder in various solvents. **a.** Comparison of all pure solvents tested. **b.** Isopropanol (IPA). **c.** Water (H_2O). **d.** Methanol (MeOH). **e.** *N*,*N*-dimethylformamide (DMF). **f.** *N*-methylpyrrolidone (NMP).

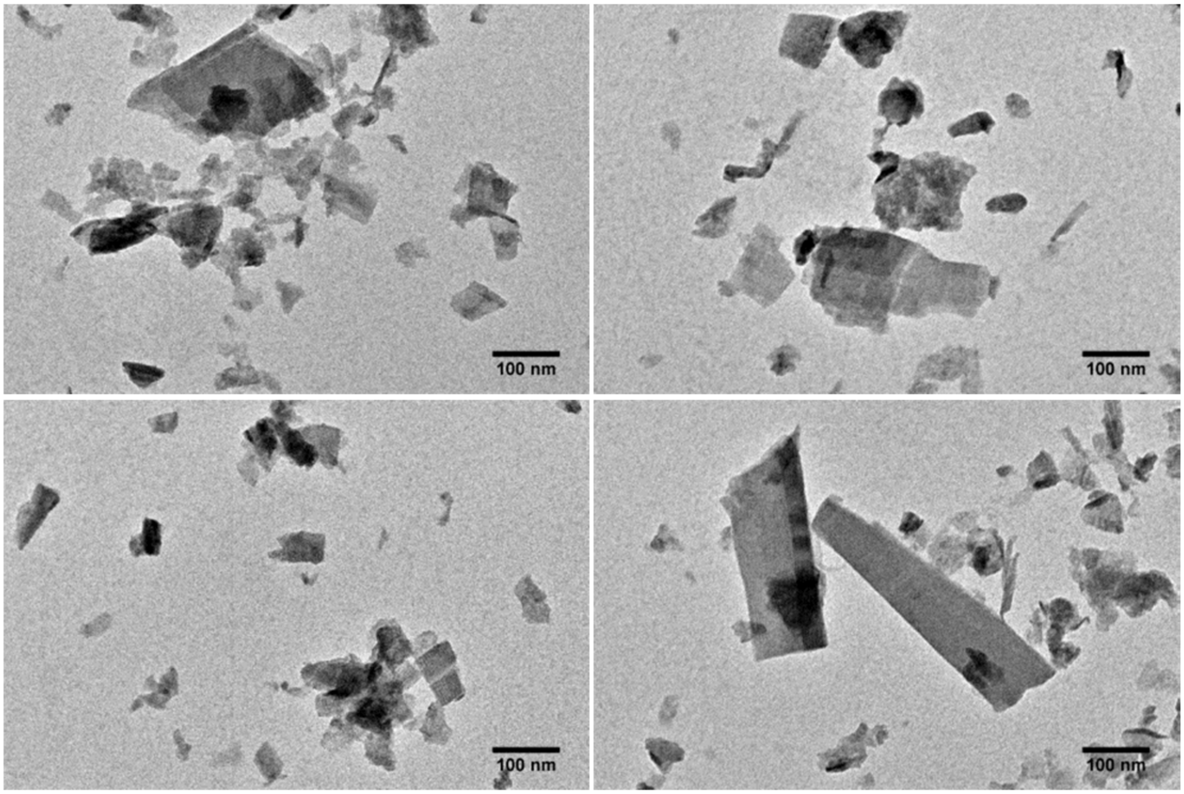

Supplementary Figure 28 | TEM images of franckeite nanosheets obtained in methanol from a 1 mg·mL^{-1} powder dispersion. Nanosheets coming from the colloidal suspension in methanol resulting from the centrifugation of the sonicated sample.

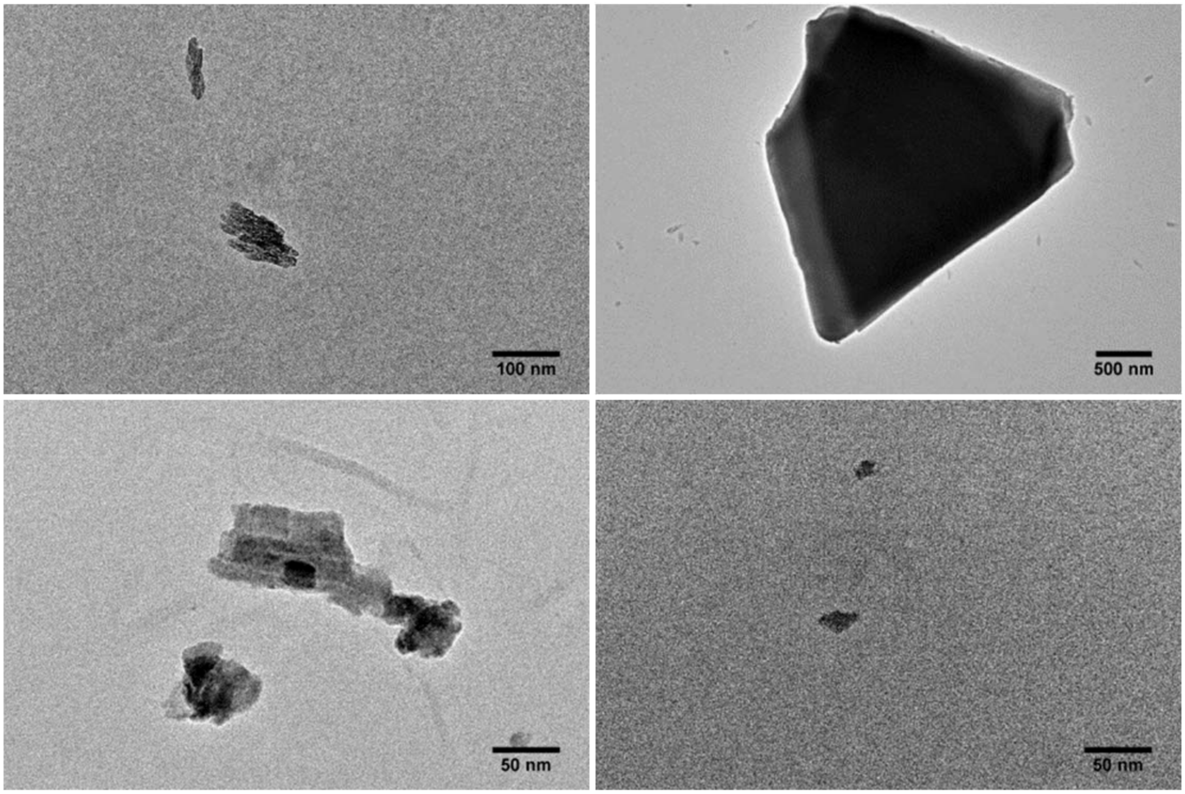

Supplementary Figure 29 | TEM images of franckeite nanosheets obtained in *N*,*N*-dimethyl-formamide (DMF) from a 1 mg·mL^{-1} powder dispersion. Nanosheets coming from the colloidal suspension in DMF resulting from the centrifugation of the sonicated sample.

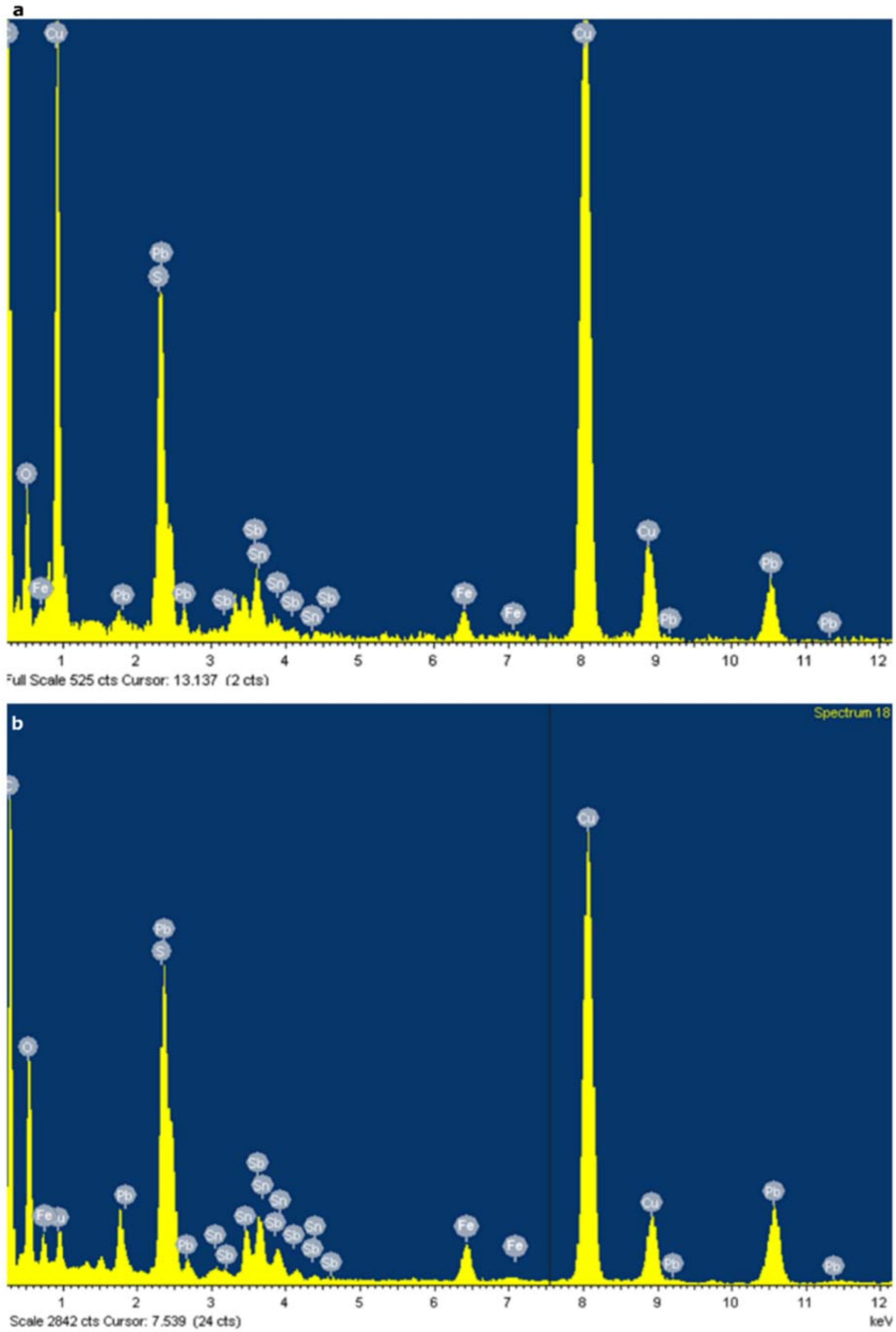

Supplementary Figure 30 | EDX spectra of exfoliated franckeite nanosheets. a, obtained in NMP from a 100 mg·mL^{-1} powder dispersion; **b**, obtained in IPA/water 1/4 from a 1 mg·mL^{-1} powder dispersion.

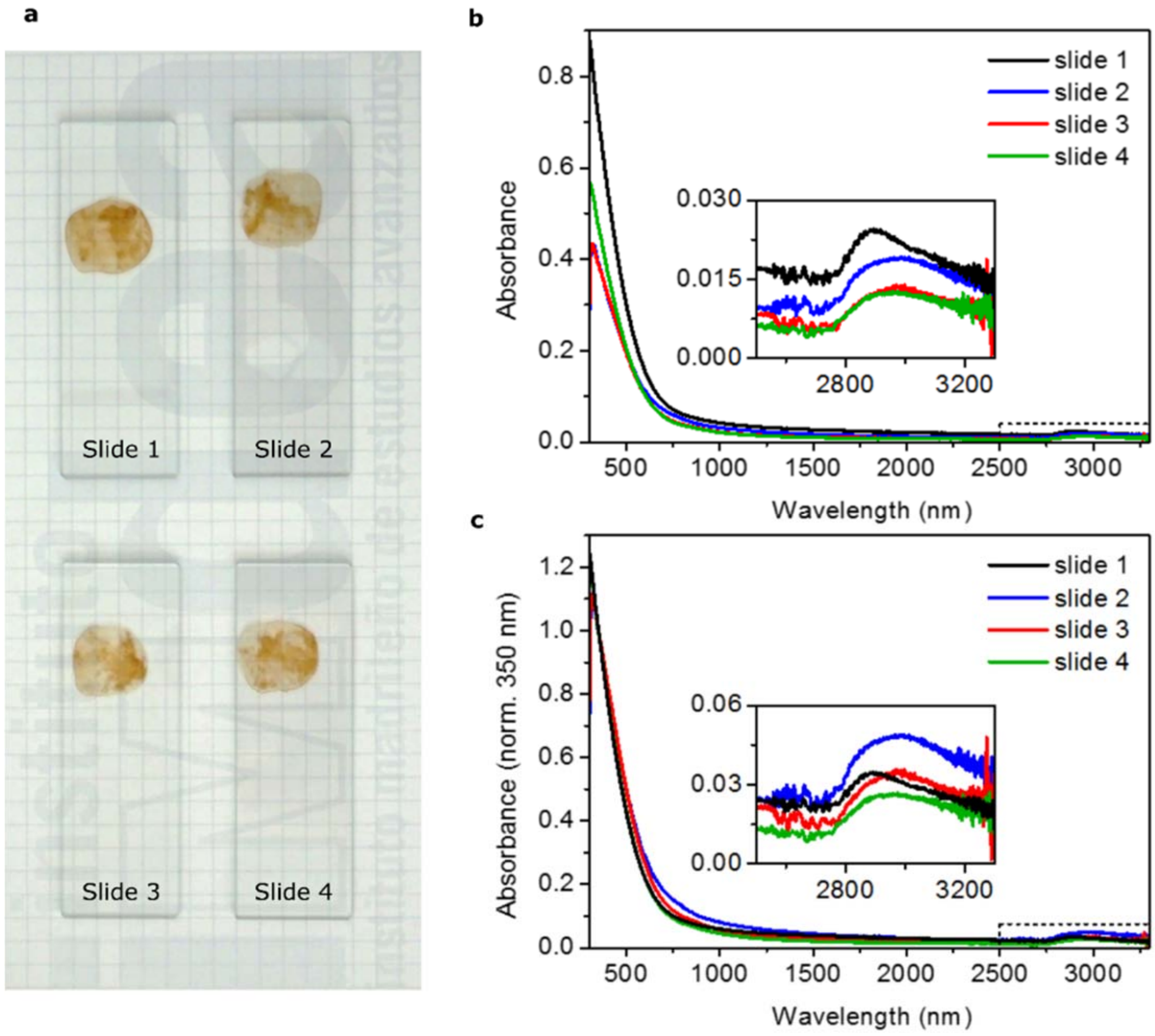

Supplementary Figure 31 | UV-Vis-NIR spectra of exfoliated franckeite (obtained from the 100 mg.mL^{-1} powder dispersion) drop-casted and dried onto glass slides. a, Exfoliated sample films deposited onto the four different glass slides used for the measurements. **b**, Absorption spectra of the sample shown in the main text (slide 1, black line), and of the three replicates performed with other three different glass slides (slides 2, 3 and 4; blue, red and green lines respectively); inset: zoom in the 2500 nm-3300 nm region. **c**, Absorption spectra normalized at 350 nm for the four slides presented in **c**; inset: zoom in the 2500 nm-3300 nm region.

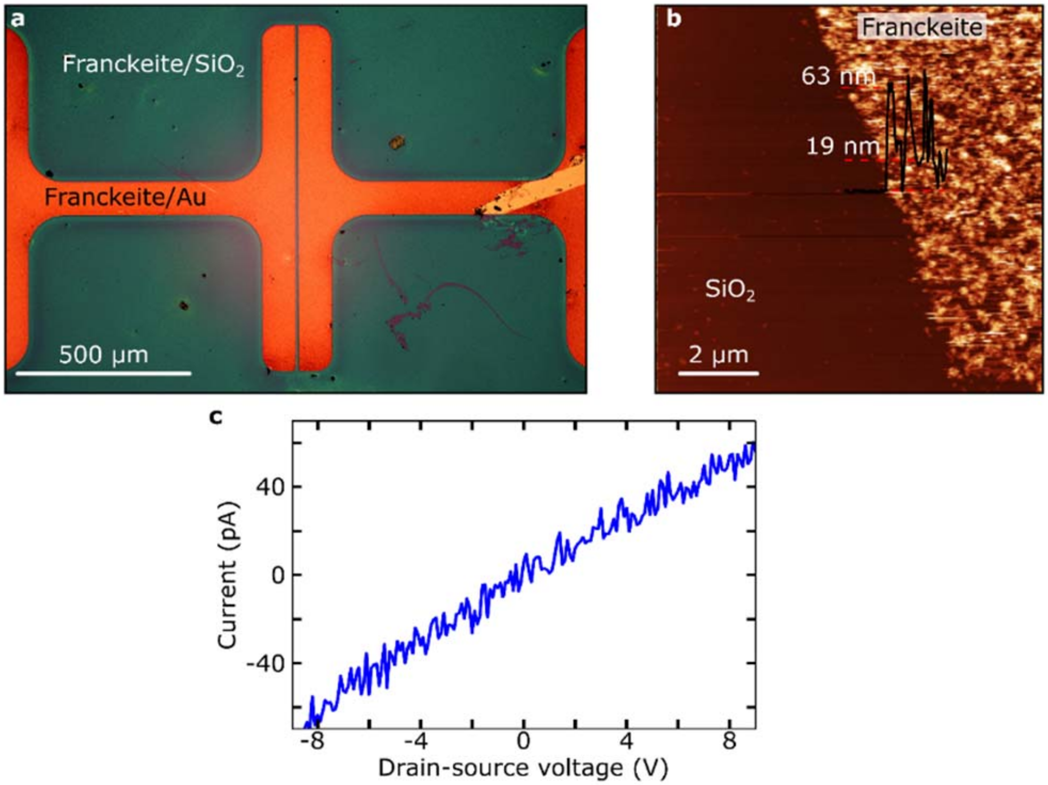

Supplementary Figure 32. a, Optical microscopy image of the device based on liquid-phase exfoliated franckeite fabricated by drop casting a suspension of franckeite in NMP on Au electrodes pre-patterned on a SiO_2 substrate. **b**, AFM topographic characterization of the franckeite layer of the device shown in a. The thickness of the layer ranges between 19 nm and 63 nm. **c**, Electronic characterization of the device in dark conditions.

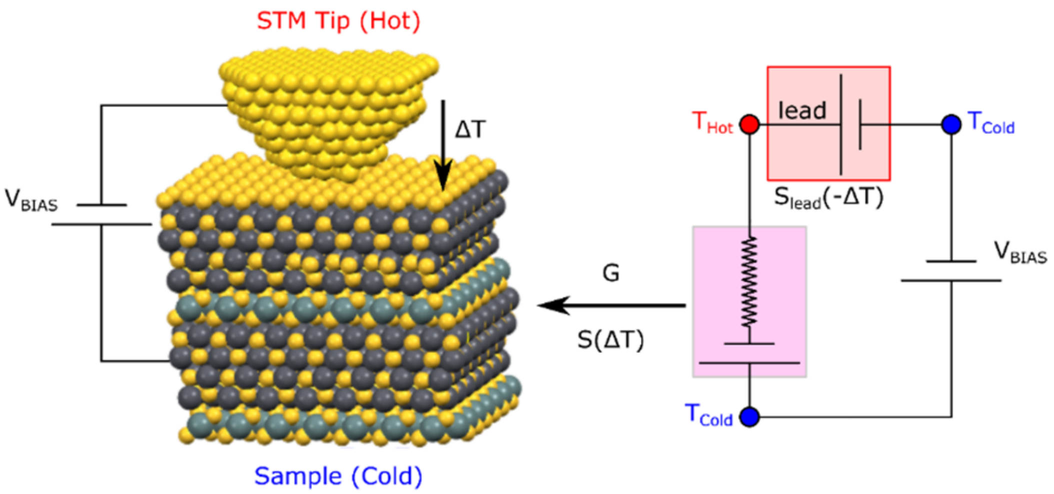

Supplementary Figure 33 | Scanning tunneling microscope for measuring thermopower. Equivalent thermal circuit of the setup for measuring the thermopower. The sample is kept at ambient temperature T_{Cold} while the tip is heated to a temperature of $T_{\mathrm{Hot}} = T_{\mathrm{Cold}} + \Delta T$. S is the thermopower of franckeite and S_{lead} is the thermopower of the tip connecting lead. V_{BIAS} is the bias voltage applied at the sample.

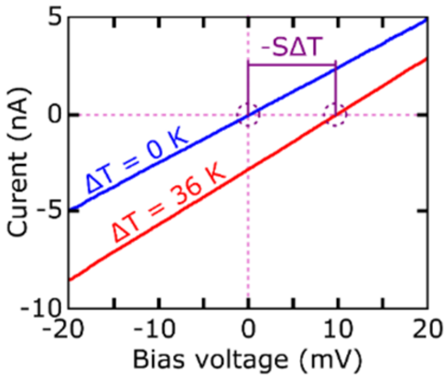

Supplementary Figure 34. Current-voltage curves acquired on franckeite at $\Delta T = 0$ K (blue) and $\Delta T = 36$ K (red) showing the thermal voltage $-S\Delta T$ due to the temperature gradient in the sample.

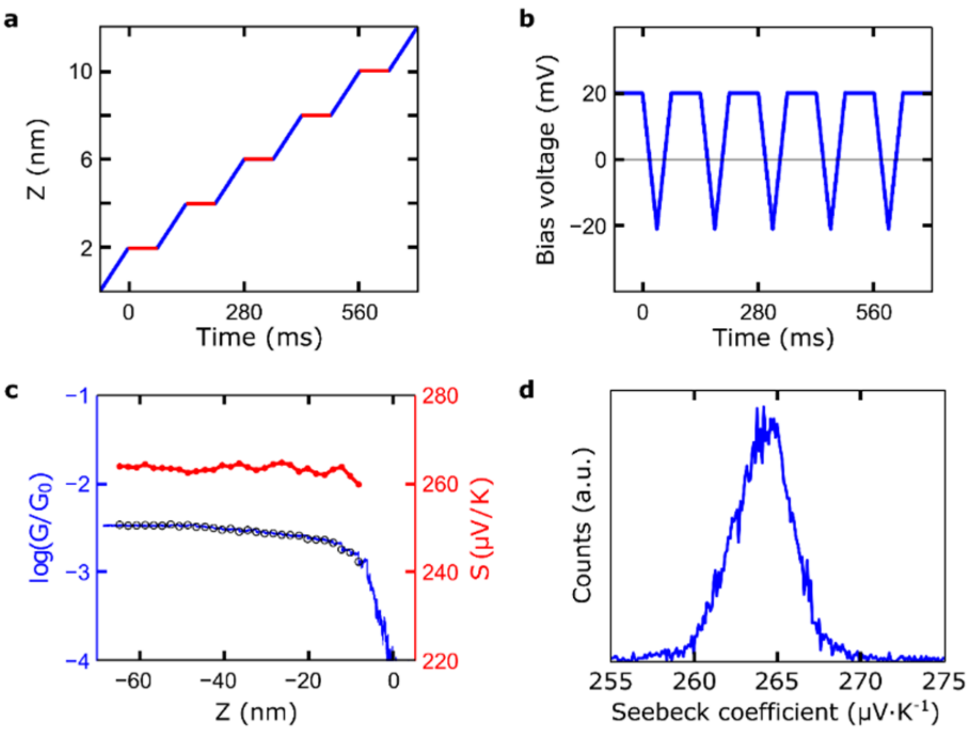

Supplementary Figure 35 | Measurement of the thermopower. a and **b,** Tip displacement z and applied bias voltage V_{BIAS}, respectively, as a function of time. The bias voltage is maintained at a fixed value 20 mV during the tip motion and every 2 nm it is swept between ±20 mV while the tip is stationary (examples of *IV* curves are shown in Fig. S34). **c,** Characteristic example of conductance (blue) and thermopower (red) acquired simultaneously for approach of the Au tip and large contact formation with the franckeite. The temperature gradient across the junction was set to $\Delta T \approx 36$ K. Note that the thermopower and conductance stabilizes when a large contact is established, indicating that indeed the thermopower value measured is the bulk value. Black open circles indicate the points where the tip motion stops and the current-voltage curves were recorded. **d,** Histogram of thermopower of franckeite, out of 85 traces at $\Delta T \approx 36$ K.

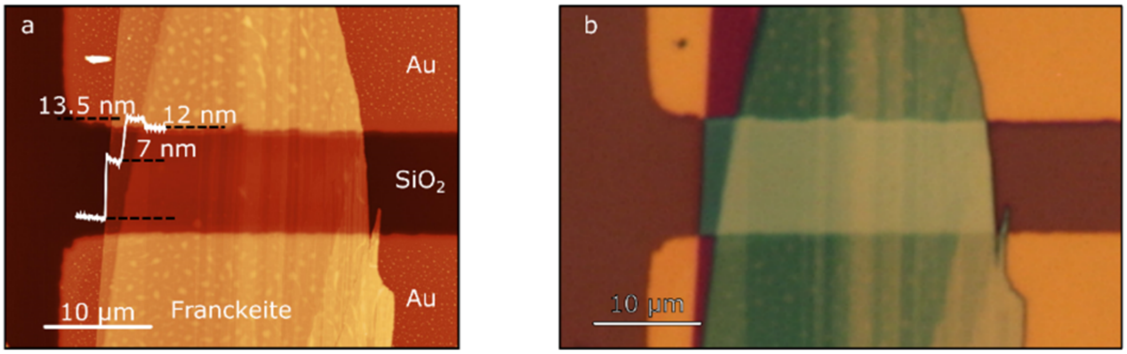

Supplementary Figure 36. a and **b,** AFM and optical microscopy image of the franckeite device characterized in Fig. 4 of the main text, respectively.

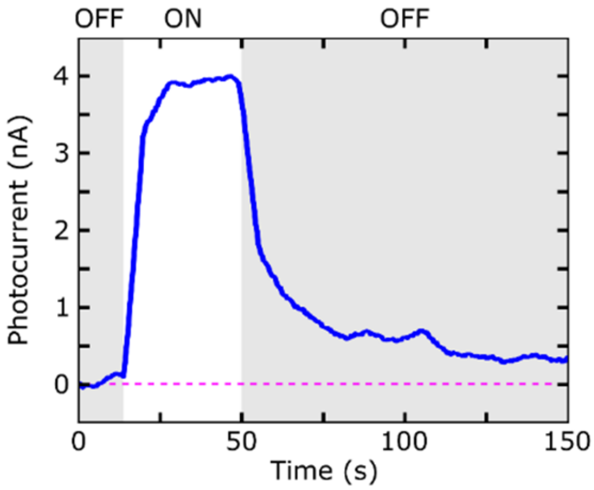

Supplementary Figure 37. Photocurrent as a function of time of the device characterized in Fig. 4 of the main text. The rise time is ~ 10 s, while the fall time is > 100 s.

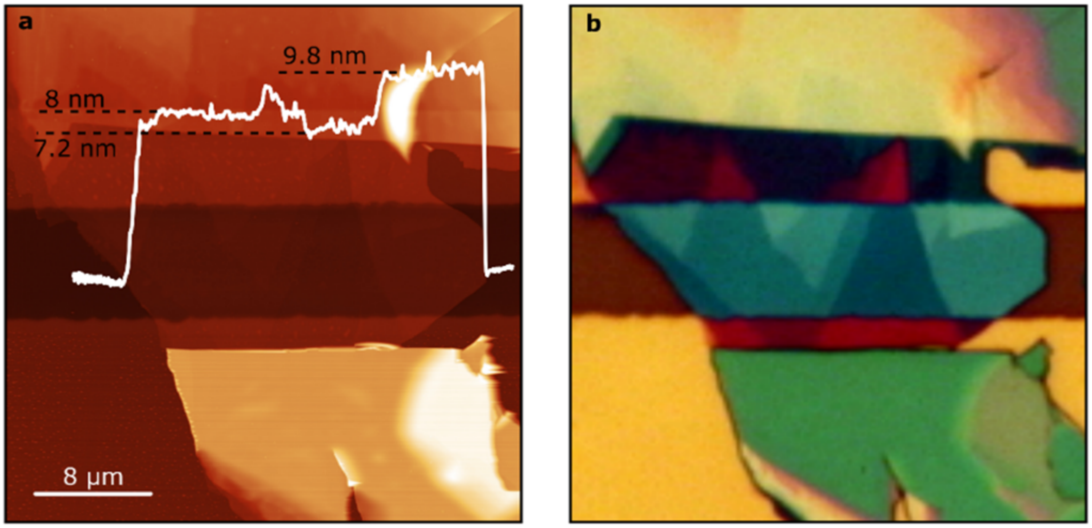

Supplementary Figure 38. a and **b,** AFM and optical microscopy image of a franckeite device, respectively.

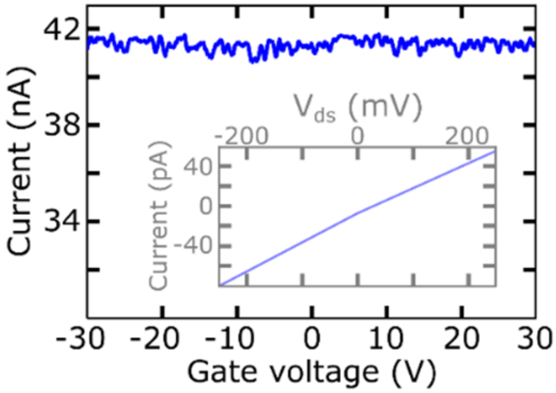

Supplementary Figure 39. Drain-source current measured as a function of the applied gate voltage of the franckeite-based device in dark conditions (V_{ds} = 1 V). Inset: current-voltage characteristics of the device measured with an applied gate voltage of V_g = 40 V.

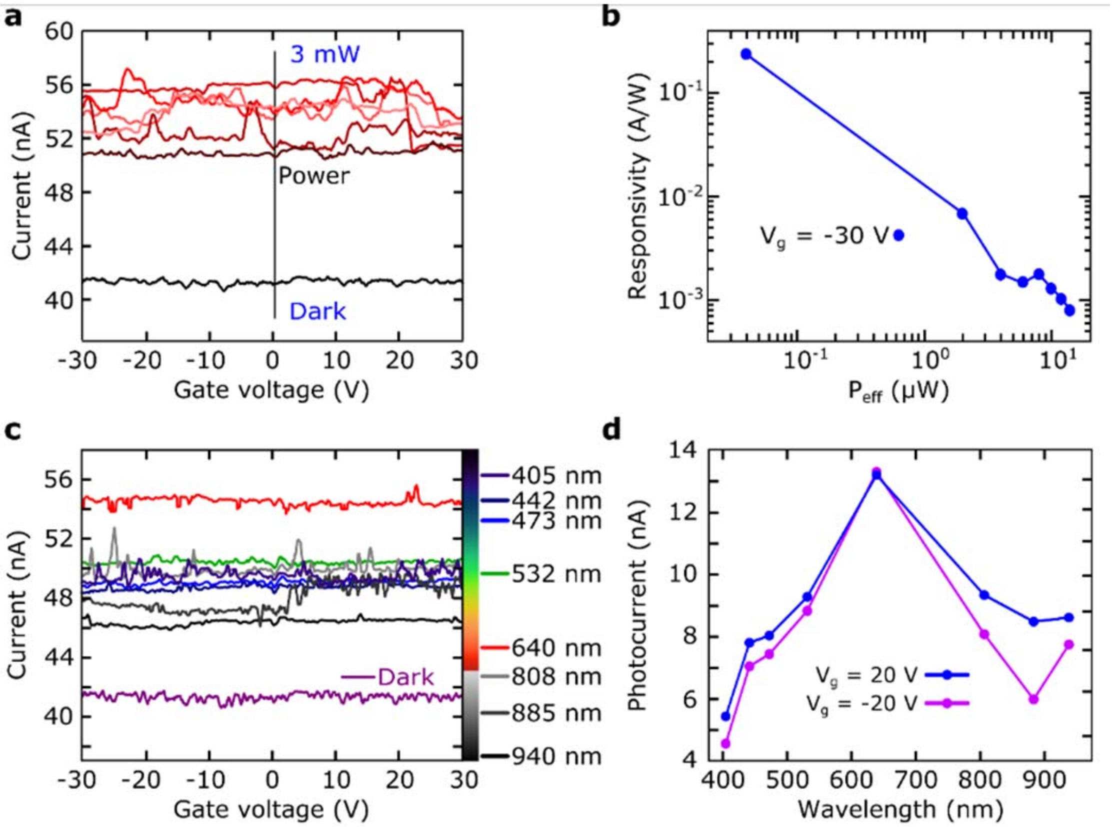

Supplementary Figure 40. a, Current as a function of the applied back-gate voltage (V_{ds} = 150 mV) for the device shown in **a** in dark conditions and upon illumination with a 640 nm wavelength laser with different powers. **b**, Responsivity of the device shown in a upon illumination with a 640 nm wavelength laser as a function of the laser effective power with an applied back-gate voltage of V_g = -30 V and V_{ds} = 150 mV. **c**, Current as a function of the applied back-gate voltage (V_{ds} = 150 mV) for the device shown in **a** upon ilumination with lasers of different wavelengths at the same intensity (P_d = 6.3 mW·cm^{-2}). There is photocurrent generation even at wavelengths as large as 940 nm. **d**, Photocurrent as a function of the laser wavelengths with the same light intensity for back gate voltages of -20 V and +20V and V_{ds} = 150 mV.

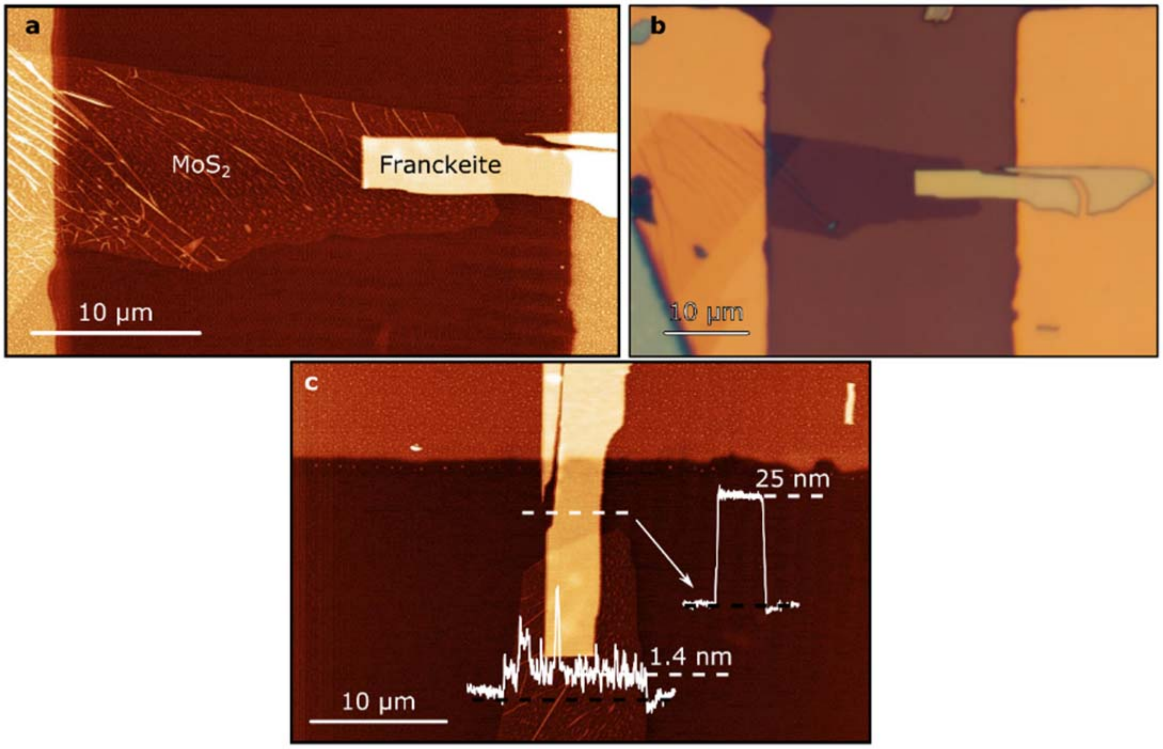

Supplementary Figure 41. a and **b,** AFM and optical microscopy image of the p-n junction (MoS_2 - franckeite) characterized in Figure 5 of the main text. **c**, AFM topographic image showing the height profile of both flakes, i.e., MoS_2 and franckeite.

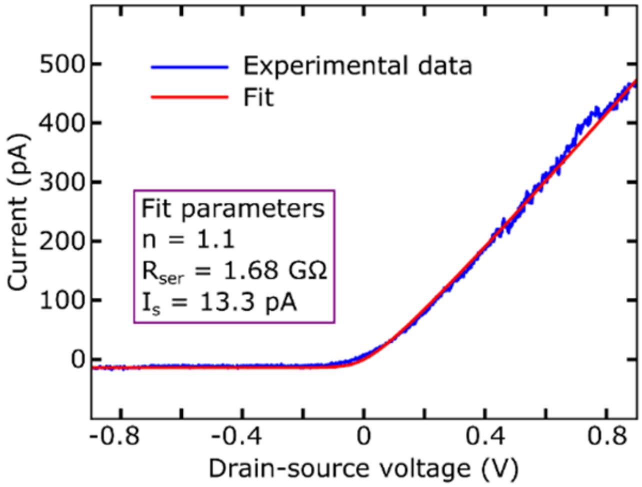

Supplementary Figure 42. Current-voltage characteristics in dark and $V_g = 0$. The experimental data (blue) have been fit to a Shockley diode model including parasitic resistance (red), obtaining the fitting parameters listed in the box.

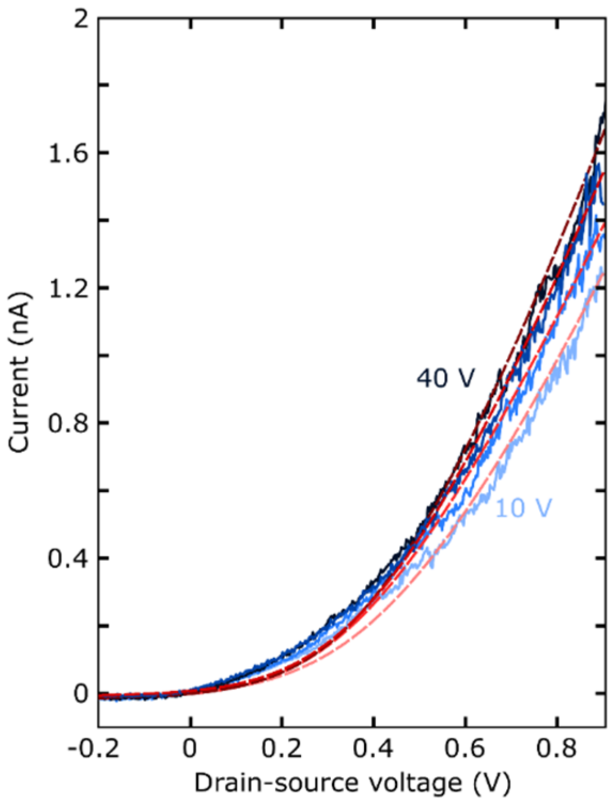

Supplementary Figure 43. Current-voltage characteristics in dark at increasing back-gate voltage (from 10 V to 40 V). The experimental data (from light blue to dark blue) have been fit to a Shockley diode model including parasitic resistance (from light red to dark red).

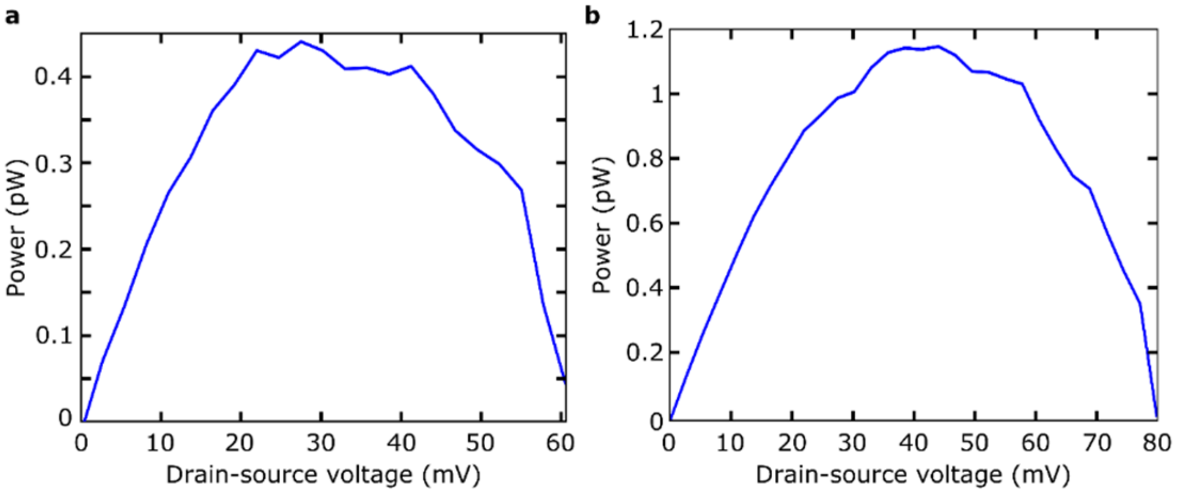

Supplementary Figure 44 p-n junction electrical power harvested in the device, calculated as $P_{el} = |I_{ds}| \cdot V_{ds}$ upon illumination with a laser spot of **a** 940 nm wavelength with a $P_{el,max}$ ~ 0.5 pW and **b** 885 nm wavelength with a $P_{el,max}$ ~ 1.2 pW.

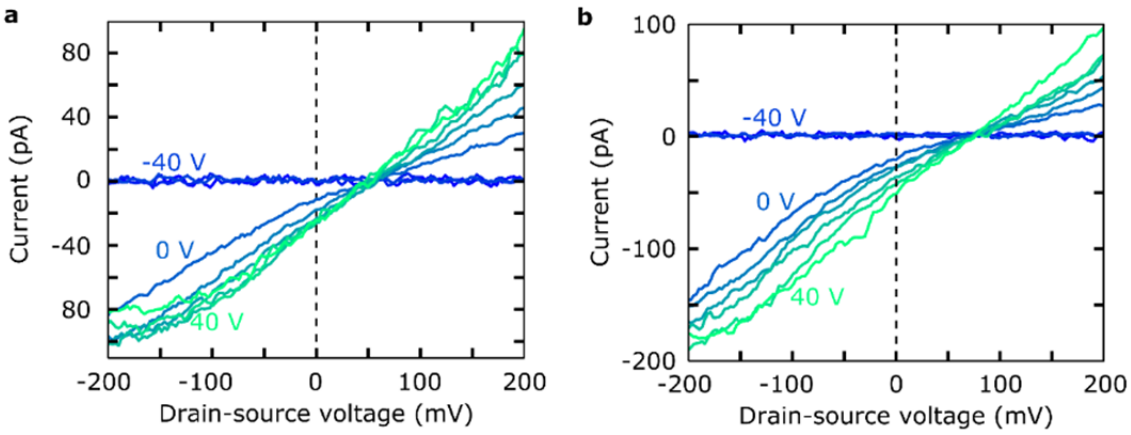

Supplementary Figure 45. p-n junction current-voltage characteristics for different back-voltages ranging from -40 V to 40 V upon illumination with **(a)** 940 nm wavelength and **(b)** 885 nm wavelength.

Supplementary Tables

Supplementary Table 1. Fundamental band gaps (in eV) of H layer, Q layer, franckeite crystal and franckeite with 50% Sn and 50% Sb in the Q layer and 100% Sb in the H layer.

	GGA-PBE	GGA-PBE + SOC	HSE06
SnS_2	0.95	0.95	2.00
PbSnS	0.50	0.30	0.70
Franckeite	0.50	0.35	0.75
Franckeite (Q-Sb/Sn and H-100% Sb)	0.20	0.03	0.50

Supplementary Table 2. Diffraction peaks of the XRD pattern.

***2θ* (º)**	**Counts**	**hkl Ref.[1]**
10.2215	230	002
15.3530	215	003
20.5132	877	004
25.7355	2701	005
30.9960	1957	020
33.8352	46	-
36.3505	40.72	-
37.0024	66	-116/-213
38.4193	66	204
39.0037	40	116
40.1041	50	-
40.4206	67	025
41.7489	152	008
43.7545	90	-
47.3494	157	-
50.0648	71	-

58.6927	79	00.11
64.6646	129	00.12
70.6909	49	-
98.5968	45	00.17

Supplementary Table 3. Experimental binding energies for the mineral chips (Min) and the single flake (Flk) of franckeite.

	Sb $3d_{3/2}$	Sb $3d_{5/2}$	Sn $3d_{3/2}$	Sn $3d_{5/2}$	S $2p_{1/2}$	S $2p_{3/2}$	Pb $4f_{5/2}$	Pb $4f_{7/2}$
A-doublet $_{Min}$	538.7	529.4	494.3	485.9	162.2	161.0	142.60	137.7
B-doublet $_{Min}$	539.8	530.4	495.2	486.9	163.0	161.8	143.5	138.6
A-doublet $_{Flk}$	539.3	530	494.3	485.9	162.8	161.4	142.8	138.0
B-doublet $_{Flk}$	540	531.5	494.9	486.9	163.4	162.2	143.8	139.0

Supplementary Table 4 | Interpretation of Raman spectra of franckeite (powder and exfoliated particles).

Raman shift (cm^{-1})	Phonon mode attribution	Homogeneous material reference			
		Raman shift (cm^{-1})	Compound	State	Literature citation
66	SA	68	PbS	nanocrystal	2, 3
	2TA	73		$(PbS)_{1.18}(TiS_2)_2$ misfit	4
145	2nd order effect	131	SnS_2	nanosheets	5
		135		nanocrystallites	6
		140.5		bulk	7
	TA + TO	151	PbS	$(PbS)_{1.18}(TiS_2)_2$ misfit	4
		154		bulk single crystal	8
194	LO(Γ)	203	PbS	$(PbS)_{1.18}(TiS_2)_2$ misfit	4
		204		bulk single crystal	8
		215		nanocrystal	2, 3
	E_g	198	SnS_2	nanocrystallites	6
		205		bulk	7
		212		nanosheets	5
		215		film (CVD)	9
253	combination		PbS + SnS_2		4
318	A_{1g}	309	SnS_2	nanocrystallites	6
				nanosheets	5
		312		film (CVD)	9
		315		bulk	7
400-650	2LO(Γ)	412	PbS	$(PbS)_{1.18}(TiS_2)_2$ misfit	4
		415		nanocrystal	3
		454		bulk single crystal	8
	3LO(Γ)	630		nanocrystal	3
		632		$(PbS)_{1.18}(TiS_2)_2$ misfit	4
	2nd order effect	450-650	SnS_2	nanocrystallites	6
				nanosheets	5

Abbreviations: phonon modes TO: transverse optical; TA: transverse acoustic; LO: longitudinal optical; SA: spheroidal acoustic. (Γ): Γ point of the Brillouin zone.

Supplementary Notes

Supplementary Note 1. Density functional theory calculations of thickness dependent band structure

Supplementary Figure 1 shows the evolution of the calculated band structure with the number of layers. It is found that the band structure topology and the bandgap was not significantly changed with a thickness from 1L to 3L, being essentially preserved up to the bulk calculation with an infinite number layers.

Supplementary Note 2. Density functional theory calculations of the band structure with SOC

As a heavy element such as Pb is present in the Q layers, relativistic effects are expected to be large and have an appreciable effect on the electronics properties. To account for such relativistic effects, spin-orbit coupling (SOC) is here included in the calculations with the GGA functional. For the H layers, as shown in Supplementary Figure 2a, the inclusion of SOC did not change the band structure significantly, and, consequently, the band gap. On the other hand, in the case of Q layers and franckeite crystal (see Supplementary Figure 2b and 2c), the band gap has slightly decreased when SOC is taken into account. To be precise, as shown in Supplementary Table 1, the direct gap at X has decreased by 0.20 eV in the PbSnS structure (Q layer) and the band gap at X has decreased by 0.15 eV in the franckeite structure, almost closing the gap.

Supplementary Note 3. Density functional theory calculations of band structure with HSE06

Since GGA-PBE functionals usually underestimate the band gaps, we use here the hybrid nonlocal exchange-correlation functional (HSE06) in an attempt to get more reliable values, possibly closer to the experimental ones. The results without SOC are given in Supplementary Table 1. The improvement in all cases is evident. The increased band-gap more than compensates the decrease due to SOC, given an overall finite gap for the franckeite crystal with and without Sb doping.

Supplementary Note 4. Density functional theory calculations of Sb-doped franckeite

In Fig. 1 of the main text we show the density functional theory calculations of the band structure of franckeite considering that the crystal is composed of only PbS and SnS_2 layers. Nevertheless, it is found that the composition of franckeite includes Sb substitutional atoms replacing Sn atoms which may donate one more electron to the system as a cation in the H layer. We compute the calculations for a 50% and 100% substitution, i.e., 50% (or 100%) of the Sn atoms have been replaced by Sb atoms in the H layer. In Supplementary Figure 3a we show the calculated band structure of franckeite composed only of PbS and SnS_2 (it is also shown in Fig. 1d of the main text), where we find a bandgap of ~ 0.4 eV with the Fermi level lying below the valence band. When we introduce an Sb substitution of 50% of (Supplementary Figure 3b), one of the valence bands becomes almost fully occupied, while for a 100% substitution (Supplementary Figure 3c) both bands are essentially full, leaving a small concentration of holes in the system. The gap has been

closed at this stage, but this can be attributed to the known deficiencies of the standard approximation to the density functional that we have used and we expect it to remain open in reality. Substitutional Sb in the Q slab or substitutional Fe in the H layer, as well as an increase in the percentage of Pb in the Q slab, does not fundamentally change the spatial separation of electrons and holes in the heterostructure. The saturation with Sb in the H layer, maybe beyond nominal concentration values, seems to be determinant for the semiconducting behavior to emerge, but this coincides with the strong presence of Sb^{4+} observed in our samples by micro-XPS and EDX.

Now we proceed to investigate the case in which the substitutional Sb atoms appear in both the Q and the H layer. We fix the amount of Sb in the Q layer at a substitution of 50% of the Sn atoms, while we change the amount of Sb from 0% (Supplementary Figure 4a), 50% (Supplementary Figure 4b) and 100% (Supplementary Figure 4c) in the H layer. Here we observe that the amount of Sb doping is crucial to open a gap in the band structure and, in the situation of 50% Sb, 50% Sn in the Q layer and 100% Sb in the H layer, a minimum bandgap of ~ 0.2 eV appears. These results confirm that, although these calculations are simple approaches to the actual franckeite structure, the presence of Sb in the franckeite composition is changing the band structure in such a way that it opens a small bandgap and provides a p-doping. As expected, the inclusion of SOC closes the band gap for the case of 50% Sb and 50% Sn in the Q layer and 100% Sb in the H layer, as shown in Supplementary Figure 5b. This gap closing should, however, be compensated with the use of HSE06 functional.

Supplementary Note 5. Scanning tunneling microscopy characterization

Bulk franckeite material was investigated by means of scanning tunneling microscopy (STM) and spectroscopy (STS). For this purpose, a piece of franckeite (Supplementary Figure 6a) is placed on a Au substrate which serves as electrode (indium is used to ensured electrical contact between the two of them), and a mechanically cut Au wire is used as a tip. Franckeite is then exfoliated to obtain a clean surface. Supplementary Figure 6b shows a STM topographic image of the franckeite surface taken with an applied bias voltage of V_{bias} = 1 V and at room temperature and ambient pressure, where a step-edge can be identified with a height of ~ 2.3 nm (~ 1 or 2 layers, depending on if the top layer is a SnS_2 or a PbS layer). The average of a set of 200 STS current-voltage characteristics is shown in Supplementary Figure 6c, depicting a clear semiconducting *p-doped* behavior: there is a zero conductance zone around zero bias voltage which extends between two well-defined values, corresponding to the valence band (B_{V}) and conduction band (B_{C}) of the material; the doping of the material can be determined from the fact that the curve is "shifted to the left", meaning that the Fermi level (corresponding to the zero bias voltage) is closer to the valence band than to the conduction band. The whole set of 200 current-voltage curves is shown in the inset in logarithmic scale where the B_{V} and B_{C} values can be extracted, obtaining B_{V} = -0.25 eV and B_{C} = 0.35 eV, yielding and electronic bandgap of $E_{\mathrm{g,el}}$ = 0.6 eV. At this point, the thermal broadening of the bands due to the temperature of the sample must be considered in the calculation, adding Et ≈ $3.5k_{\mathrm{B}}T$ = 90 meV at room temperature,[10] therefore, the electronic bandgap of franckeite is $E_{\mathrm{g,el}} \approx 0.7$ eV.

Supplementary Note 6. X-ray diffraction characterization

Francheite powder was studied by means of X-ray diffraction in a X'pert PRO θ/2θ diffractometer (Panalytical) at room temperature in the facilities of Servicio Interdepartamental de Investigación (SIdI), Universidad Autónoma de Madrid. The measured spectra is shown in Supplementary Figure 7, together with a fit for each peak. The diffraction peaks are in good agreement with previously reported values (listed in Supplementary Table 2),[1] resembling a P2/m space group.

Supplementary Note 7. XPS on franckeite mineral chips

In addition to the local spectroscopy on a single flake in the main text we have performed the chemical characterization of the franckeite mineral with standard XPS, using the Al Kα line from a conventional monochromatized X-ray source described elsewhere[11]: several fragments of a few millimeters in size of mineral chips covering a platinum surface were analyzed. There are no photoemission data in the literature about franckeite, but the overview photoemission spectrum (not shown) of the mineral gives the atomic components and contaminants of the franckeite surface: Sb, Sn, Pb and S, as well as the other typical minor components of franckeite Fe and Ag,[12, 13, 14] appearing as contaminants oxygen and carbon. The XPS narrow scans give information on the oxidation state and the possible adscription to the different structural layers of the franckeite.

In the minor components of franckeite, iron is present in a very small concentration, although it is apparent in the Sn $3p_{3/2}$ narrow scan (Supplementary Figure 10a). The broad shape and the position of Fe $2p_{3/2}$ at 708 eV in binding energy, and the distance of 13.8 eV from Fe $2p_{1/2}$, suggest Fe^{2+} or Fe^{3+} valence state similar to some iron sulfides (Fe_7S_8, FeS_2).

Silver is typical in cylindrite group of minerals like incaite and potosiite, and it is present in this sample in a small amount too: Ag $3d_{5/2}$ core level (Supplementary Figure 10b) appears at 367.8 eV binding energy that could be compatible with Ag inclusions in an oxide state. As contaminants we find carbon and oxygen: C 1s peak at 285.0 eV; oxygen core level O1s is not directly observable due to its coincidence with Sb 3d that it is one of the most intense peaks, but the oxygen Auger KLL transition is visible in the overview spectrum. After fitting the components for Sb 3d core level (Supplementary Figure 11) the O1s appear under Sb 3d5/2 as a broad (fwhm of 2.75 eV) peak at 531.5 eV, indicating several oxygen states.

XPS narrow spectra of Sb 3d, Sn 3d, S 2p and Pb 4f regions of mineral franckeite are shown in Supplementary Figure 11. The fits to these spectra are generally satisfactory, with the exception of the poor fit at the low binding energy tail on the Sb 3d and Sn 3d spectra. The corresponding binding energies extracted from the fits are shown in Supplementary Table 3 and compared to the binding energies of the same components for the single flake experiment measured with synchrotron radiation technique. In the last case we measured a shift towards higher binding energy of 0.3 – 0.4 eV for the low binding energy components (S and Pb measured at $h\nu$ = 230 eV), and 0.6 – 1.0 eV for Sb (measured at $h\nu$ = 600eV), with a good agreement for the case of Sn. As we do not expect different chemical species in both cases, because the flake comes from the same mineral fragment, we attribute these shifts towards higher binding energies to a charging effect due to the much higher photon flux in the synchrotron radiation experiment than in the conventional Al Kα x ray source (depending on the energy shift in the particular photon flux).

As in the single flake, the best fit with the minor number of meaningful components results in two doublets for each of the measured element: we name “A-doublet” to the doublet with lower binding energy and “B-doublet” to the one with higher binding energy. The A-doublet of Pb 4f (with Pb $4f_{7/2}$ at 137.7 eV) corresponds to the lead in the Q layer containing PbS,[15, 16] while the B-doublet (Pb $4f_{5/2}$ at 138.6 eV) can be attributed to a lead oxide component. In the Sn 3d core level, the A-doublet (Sn $3d_{5/2}$ at 485.9 eV) can be attributed to the state Sn^{2+}, and the B-doublet (Sn $3d_{5/2}$ at 486.9 eV) to Sn^{4+}: the difference in near 1 eV between A- and B-doublet is in accordance with the literature for these states.[15, 17, 18, 19] But in the franckeite structural model[12] the Sn^{2+} ion is a minority respect Sn^{4+} and it is present in the Q layer while the Sn^{4+} ion is majority and present in the H layer as SnS_2, whereas in our sample the Sn^{2+} component is pretty much higher than Sn^{4+}. The fact that we see more amount of Sn^{2+} than Sn^{4+} can indicate that fracturing/exfoliation occurs preferentially leaving exposed the Q layer, explaining at the same time the appearance of the high binding energy component of Pb as surface atoms with a different chemical shift or a lead oxide component. The Sb as well can be fit too with two doublets, although there is a poor quality at the low binding energy side of Sb $3d_{5/2}$ that can indicate a surface state or defects produced by the fracturing. The B-doublet (Sb $3d_{5/2}$ at 530.4 eV) corresponds to Sb^{3+} state in accordance with literature,[20] but the A-doublet (Sb $3d_{5/2}$ at 529.4 eV) that has lower binding energy and a high intensity must be attributed to Sb inclusions or substitutional defects. For S 2p, the A-doublet (S $2p_{3/2}$ at 161 eV) and the B-doublet (S $2p_{3/2}$ at 161.8 eV) are completely in accordance with the lead and tin sulfide.

From the area of the most intense peaks and the atomic sensitivity factor we can estimate the composition as $Pb_{25.6}Sn_{9.0}Fe_{0.7}Sb_{10.0}S_{53.7}Ag_{1.0}$. This is close to the composition found by

Makovicky *et al.*[14] of $Pb_{21.7}Sn_{9.3}Fe_{4.0}Sb_{8.1}S_{56.9}$ but with some discrepancies, appearance of extra Sb in our samples, and the presence of Ag in higher amount than Fe.

AFM characterization of mechanically exfoliated flakes for micro-XPS

In Supplementary Figure 12b and c we show AFM topographic images of the mechanically exfoliated flake used to perform the micro-XPS measurements (Supplementary Figure 12a).

Supplementary Note 8. Liquid-phase exfoliation

All the UV-Vis-NIR spectra of this section have been performed on colloidal suspensions in a quartz cuvette (path: 1 cm) using a Cary 5000 spectrophotometer (Agilent Technologies). Due to particle size, most of the colloids present non-negligible scattering, consequently the spectra shown are extinction spectra that sum both absorbance and scattering of the samples.

Liquid-phase exfoliation in NMP

We direct the reader to Fig. 3 in the main text and Supplementary Figures 13 to 16 for information about the Liquid-phase exfoliation in NMP.

Liquid-phase exfoliation in IPA/water mixtures

Exfoliation experiments in various IPA/water mixtures (1/0, 4/1, 1/1, 1/4, 0/1) were carried out as described in the "Methods" section: 1 h bath sonication of 1 mg·mL^{-1} franckeite dispersions in the chosen mixture, followed by centrifugation to get a colloidal suspension of franckeite nanosheets in the proper mixture. Optical images and extinction spectra of the corresponding samples show that only IPA, the 1/1 and the 1/4 mixtures are able to both produce and disperse exfoliated nanosheets, the latter to a lesser extent (Supplementary Figure 17a, 18a).

To better compare and precise the exfoliation efficiency within the series, the sediments originating from the sonication-centrifugation sequences of each sample were redispersed (1 min sonication) in IPA. The previous observations indeed proved that IPA is a good dispersing medium for franckeite nanosheets and particles. After centrifugation of the sediment dispersion in IPA, new colloidal suspensions are obtained that contain exfoliated material initially produced but not always properly dispersed in the original IPA/water mixtures. Figs. S14b, S18b-f and S19a show optical images and the corresponding extinction spectra of these various IPA redispersions. From these results, it appears that exfoliation is occurring in IPA/water 4/1 and water, although in the latter case scattering is dominating the extinction spectrum and the quantity of produced material is very low. Exfoliated material can also be recovered from the other samples, in similar proportions to that obtained from IPA/water 4/1. To be noticed is the particularly high amount of material recovered from the IPA/water 1/4 sediment; cumulated with the material initially dispersed in this mixture, the total amount of exfoliated material compares with that achievable in the best dispersing media (*i.e.* IPA and IPA/water 1/1; Supplementary Figure 18b, d, e and 19b). As almost no nanosheets were detectable in the 4/1 mixture and in water, the

cumulated exfoliated franckeite in those cases amounts to the material recovered in IPA, which represents, in the 4/1 mixture cas,e the half of the material produced in IPA, IPA/water 1/1 or 1/4.

To sum up, in our conditions: IPA and IPA/water 1/1 are both efficient media in terms of exfoliation of franckeite and dispersion of the resulting nanosheets; on the contrary, IPA/water 4/1 and water do not disperse franckeite nanosheets and produce relatively weak amount of exfoliated material; as a particular case, IPA/water 1/4, in spite of its substantial water content, is able to disperse noticeable quantities of nanosheets and produce a significant amount of exfoliated material. These remarkable properties most probably relate to IPA/water 1/4 specific surface tension that matches that of SnS_2.

The TEM images of the samples prepared in IPA/water mixtures (Supplementary Figure 20 to 25) deposited as colloid in the proper exfoliating solvent or as redispersed material in IPA in the relevant cases, evidence thin nanosheets that are globally very similar both in shapes and lateral sizes, relatively uniform and centered around 200 nm. Only the exfoliated material produced in the pure solvent show some distinct features: the IPA colloid includes an additional and significant amount of few-nanometer round-shaped nanoparticles (Supplementary Figure 20), whereas the few nanosheets recovered from the exfoliation in water are the only ones to exhibit such a damaged surface, with lots of defects (Supplementary Figure 25). Of particular interest is the comparison of Figs. S23 and S24 which shows that the nanosheets dispersed in the proper IPA/water 1/4 mixture or recovered in IPA from the corresponding sediment have exactly the same structure.

Liquid-phase exfoliation in pure polar solvents

LPE was carried out in methanol and DMF as well, with initial franckeite powder dispersions of 1 mg·mL^{-1}, and compared to the results obtained in pure IPA, water and NMP in the same conditions. Figs. S26 and S27 show the optical images and extinction spectra of the corresponding colloids. From their observation, we can conclude that methanol and DMF roughly produce as high as half the amount of exfoliated material obtained with pure IPA or NMP. Nevertheless, their TEM micrographs reveal nanostructures that are very different from the previous ones. The colloid prepared in methanol is composed of thin and mainly small flakes, ca. 50-100 nm in size, together with some nanosheets reaching up to 500 nm (Supplementary Figure 28). As for the DMF sample, it contains mostly a mixture of small (50-100 nm) to very small (<20 nm) objects that look like nanoparticles more than nanosheets. Due to DMF dispersing abilities, thicker and micrometric flakes are also to be found (Supplementary Figure 29).

Suspension colloidal stability and reproducibility of the experiments

Colloidal stability of the suspensions depends on many parameters, but mainly on the dispersing abilities of the corresponding solvent, the weight (related to size and thickness) of the particles, and the temperature. All of these parameters are involved in our exfoliation process and are to be considered to explain the results obtained and ensure reproducibility.

All samples prepared in NMP show long-term colloidal stability due to the strong coordination abilities of this solvent. Such a property also ensures highly reproducible

exfoliation experiments because it can compensate for small variations in the size of exfoliated nanosheets during bath sonication or in the temperature during centrifugation.

On the contrary some experiments, in IPA/water 1/4 for example, were found more difficult to replicate at the beginning, sometimes leading to particle-free supernatants after centrifugation. Nevertheless, redispersion in the same volume of a better dispersing solvent (NMP or IPA), and subsequent centrifugation (990 *g*, 30 min) consistently afforded stable colloids showing the same extinction properties as successfully prepared samples. We attributed this initially poor reproducibility in IPA/water 1/4 to the combined effects of the weaker dispersing abilities of this solvent, the large size of the nanosheets (ca. 200 nm) that greatly favors sedimentation, and an insufficient control of the temperature during the centrifugation step. Once better controlled the temperature during centrifugation, we reached the desired reproducibility. Nonetheless, due to nanosheet size, and IPA and water being respectively less and far less coordinating than NMP, the suspensions of this series suffer from lesser colloidal stability.

In the case of methanol and DMF, the control of the centrifugation temperature ensured the reproducibility of the exfoliation process. Once precipitated the micrometric heavier nanosheets (within ~24 h), the samples showed long-term stability thanks to the coordination properties of these solvents and the small flake size.

Supplementary Note 9. Powder and liquid-phase-exfoliated franckeite Raman spectra interpretation

The band at 66 cm^{-1} corresponds to an acoustic phonon mode in PbS,[2, 3, 4] whereas the band at 318 cm^{-1} is a clear signature of the A_{1g} mode in SnS_2.[5, 6, 7, 9] Other bands at 145 cm^{-1}, 194 cm^{-1}, and the 400-650 cm^{-1} shoulder are most likely due to a superposition of respective phonon modes of both PbS (combinations and overtones of acoustic and optical modes) [2, 3, 4, 8] and SnS_2 (including the E_g mode and second-order effects). [5, 6, 7, 9] As proposed in other works on misfit compounds,[4] the last band at 253 cm^{-1} could result from the combination of phonon modes of both layer types. Slight shifts are to be noted regarding the parent structures; they can be attributed to deviations in terms of structure[13, 21, 22] and composition (*e.g.* Sb and Fe identified by TEM/EDX)[23] between the idealized model and natural franckeite. In spite of its weakness, the interlayer van der Waals interaction can also cause some noticeable changes in the respective vibration properties of each lattice.[4]

Supplementary Note 10. Devices based on liquid-phase exfoliated franckeite

We have investigated functional devices based on liquid-phase exfoliated franckeite fabricated by drop casting a suspension of franckeite in NMP (1.25 ± 0.25 mg/mL) onto Au electrodes pre-patterned on a SiO_2 substrate and let it dry at room temperature. The device was then annealed at 120 °C in vacuum ($P < 10^{-1}$ bar) during 4 hours in order to remove completely the solvent and thus to improve the contact between the platelets. The device is shown in Supplementary Figure 32a, where the LPE-franckeite is covering the whole area,

even the Au electrodes. Atomic force microscopy topographic characterization (Supplementary Figure 32b) yields a thickness of the franckeite layer of ~ 60 nm.

The suspension of franckeite in NMP was prepared as explained in the main text, and the concentration in exfoliated material was determined by weighing the dried residue from a known volume of the most concentrated colloid. The nanosheets were precipitated with chloroform, the dispersion was centrifuged (18,600 g, 15 min) and the sediment was dried in vacuum until no variation in its weight could be measured.

The electronic characterization of the device in dark conditions (Supplementary Figure 32c) yields a sheet resistance of ~ 86 GΩ/sq, with a conductivity of $\sigma \sim 10^{-6}$ S·m^{-1}, which is just one order of magnitude smaller than the one obtained in inkjet-printed-based MoS_2 devices ($\sigma = 8.9 \cdot 10^{-5}$ S·m^{-1}).[24]

Supplementary Note 11. Thermopower

We have studied the thermopower of bulk franckeite by using a modified scanning tunneling microscope (STM) which allows to locally measure the thermopower of a surface. The gold STM tip, which is mechanically cut, is moved towards the sample for 70-90 nm to form a large contact. The sample is kept at room temperature while the tip is resistively heated up to a temperature gradient of ~35 K. In addition to the applied bias voltage, the temperature gradient gives rise to a voltage offset $-S\Delta T$, where S is the thermopower of franckeite (Supplementary Figure 33). This offset can be measured from a current-voltage curve by finding the voltage that makes the current zero. The slope of the current-voltage curve gives the conductance (Supplementary Figure 34).[25, 26]

By heating the tip we not only establish a temperature difference between the tip and the substrate but also a temperature gradient across the tip-connecting lead, which gives rise to an additional thermoelectric voltage. The equivalent circuit for measuring the thermopower is shown in Supplementary Figure 33. Considering the equivalent circuit, we can write $I = G\,(V_{bias}+S_{lead}\,\Delta T - S\Delta T)$, where S and S_{lead} is the thermopower of the franckeite and the tip connecting lead respectively, and $\Delta T = T_{Hot}-T_{Cold}$ is the temperature gradient, with T_{Hot} and T_{Cold} being the temperatures of the tip and franckeite, respectively. It has been shown previously that $S_{lead} = 0.05\ \mu V\cdot K^{-1}$, which is negligible in comparison with the thermopower of franckeite. In addition, the resulting value of the measured thermopower is the difference between the thermopower of gold and franckeite, but since the thermopower of gold is much smaller than the franckeite, its contribution is negligible.

To obtain the average thermopower of franckeite we collected data of 85 traces in a histogram and we found a positive value of $S = 264\ \mu V\cdot K^{-1}$, indicating that franckeite is a p-type material. In addition, the mean conductance was found to be $G = 0.0032\ G_0$ (R=4 MΩ), where G_0 is the quantum of conductance. The thermal conductivity of gold is expected much larger than that of franckeite when making a large contact, thus most of the temperature drops in franckeite itself and consequently the thermopower measured is the bulk thermopower of franckeite.[27]

Supplementary Note 12. Time response of the franckeite photodetector

In Supplementary Figure 37 we show the photocurrent generated by the franckeite device characterized in the main text as a function of time upon illumination with light wavelength

of 640 nm and a modulated intensity of 2.78 W/m^2. The rise time is ~ 10 s, while the fall time is > 100 s. This slow time response could be attributed to a strong photogating effect acting in our devices, where localized states are introduced within the bandgap of the material by impurities. Photogenerated charges can get trapped in these states and reside there for long times, hampering the electron-hole recombination and thus slowing down the time response of the device.[28, 29]

Supplementary Note 13. Characterization of another franckeite photodetector

We have performed the electronic and optoelectronic characterization of a franckeite photodetector different from the one shown in the main text. In Supplementary Figure 38 we show AFM topographic and optical microscopy images of the device with a thickness ranging from 7.2 nm to 9.8 nm.

The electronic characterization in dark conditions of the device (Supplementary Figure 39) was performed in vacuum (P < 10^{-5} mbar) and at room temperature. As can be depicted from the figure, the device presents again a weak dependence with the applied gate voltage, attributed to the high doping provided by the substitutional Sb atoms.

The device is illuminated afterwards in order to study the photoresponse. In Supplementary Figure 40a we show the I_{ds}-V_g curve of the device upon illumination with light wavelength of 640 nm and increasing light power from dark up to 3 mW, showing that the drain-source current of the photodetector increases with increasing light power over the full range of the gate voltage. The responsivity calculated from these measurements are represented in

Supplementary Figure 40b, where we see that the device is capable to achieve a maximum responsivity of 241 mA·W^{-1}.

We also study the photoresponse of the device by illuminating with light of different wavelengths (Supplementary Figure 40c), obtaining photocurrent generation for the full range (from 405 nm up to 940 nm), in good agreement with the photodetector characterized in the main text. Finally, the photocurrent is plotted in Supplementary Figure 40d as a function of the light wavelength for two different applied gate voltages.

Supplementary Note 14. MoS_2-franckeite p-n junction

In Supplementary Figure 41 we show AFM topographic and optical microscopy images of the p-n junction characterized in Fig. 5 of the main text, showing that the MoS_2 flake presents a thickness of ~ 1.4 nm, while the franckeite flake has a thickness of ~ 25 nm.

The p-n junction current-voltage characteristics have been fitted to a Shockley diode model with resistance in series (R_S) and in parallel (R_P) given by:[30]

$$I_{ds} = I_S \left[exp\left(\frac{V_{ds}-I_{ds}R_S}{nV_T}\right) - 1\right] + \frac{V_{ds}-I_{ds}R_P}{R_P}$$

where I_{ds} is the drain-source current passing through the junction, I_S is the saturation current, V_{ds} is the drain-source voltage, n is the ideality factor (with typically a value between 1 and 2) and $V_T = k_B T/e$ is the thermal voltage (with k_B the Boltzmann constant, T is the temperature and e the elementary charge). An explicit solution to Supplementary Equation 1 can be written in terms of the Lambert W-function:

$$I_{ds} = \frac{nV_T}{R_S} W\left\{\frac{I_S R_S R_P}{nV_T(R_S+R_P)} exp\left(\frac{R_P(V_{ds}+I_S R_S)}{nV_T(R_S+R_P)}\right)\right\} + \frac{V_{ds}-I_S R_P}{R_S+R_P}$$

For the drain-source current in dark at $V_g = 0$ V (Supplementary Figure 42), we obtain the values $I_S = 13.3$ pA, $n = 1.1$, $R_S = 1.68$ GΩ and $R_P \rightarrow \infty$. As shown, the experimental data fit very well to the Shockley diode model with parasitic resistance, and the extracted ideality factor (close to 1) suggests that our p-n junction works closely to an ideal Shockley diode. However, we have to consider here that Schottky barriers can be formed at the semiconductor-metal interface (MoS_2/Au and franckeite/Au), which are known to affect both the rectifying characteristics of the diodes and the resistance, this could explain the high value obtained for the series resistance.[31, 32]

We also observe by fitting the current-voltage characteristics of the p-n junction with positive applied gate voltages (Supplementary Figure 43) that the ideality factor increases with increasing gate voltage (reaching a maximum value of 4.3), suggesting a modulation of the charge densities and band alignment between the two semiconductors. Nevertheless, the transfer curve shown in the inset of Fig. 5c of the main text, in which the drain-source current increases with increasing gate voltage, indicates that the gate voltage is affecting mainly the MoS_2 flake. This situation was expected since we have shown that franckeite presents a rather weak field-effect (Fig. 4 in the main text) and, therefore, the electric field created by the gate voltage affects much more the carrier density in the MoS_2 flake than in the franckeite flake. As a consequence, the extracted value of R_S decreases with increasing gate voltage.

The photoresponse of the device is analyzed upon illumination with light wavelengths of 940 nm and 885 nm (Fig. 5d in the main text). Here, a photovoltaic behavior is observed due to the presence of a short-circuit current (I_{SC}) and an open-circuit voltage (V_{OC}), which increase with increasing photon energy due to a higher absorption of the franckeite flake. The electrical power harvested in the device, calculated as $P_{el} = |I_{ds}| \cdot V_{ds}$, is represented in Supplementary Figure 44 for illumination with both light wavelengths, obtaining maximum values of $P_{el,max} \sim 0.5$ pW for 940 nm and $P_{el,max} \sim 1.2$ pW for 885 nm. Although these values are rather low when compared to other p-n junctions fabricated from two-dimensional materials, they serve as a good starting point to the development of optimized p-n junctions using franckeite as p-type semiconductor.

The current-voltage characteristics upon illumination for different applied gate voltage are represented in Supplementary Figure 45. The value of I_{SC} increases as the gate voltage increases. For $V_g < 0$ V we do not observe photocurrent, while for $V_g > 0$ V the photocurrent increases with increasing V_g. This is probably due to both a modulation of the band alignment of the two materials affected by the gate voltage and the increase of the carrier concentration (electrons) mainly in the MoS_2 flake that might reduce the carrier lifetime and give rise to enhanced recombination of photogenerated carriers.[33]

Supplementary References

1. Mottana A, Fiori S, Parodi GC. Improved X-Ray Powder Diffraction Data for Franckeite. *Powder Diffraction* 2013, **7**(2)**:** 112-114.

2. Krauss TD, Wise FW. Coherent Acoustic Phonons in a Semiconductor Quantum Dot. *Physical Review Letters* 1997, **79**(25)**:** 5102-5105.

3. Krauss TD, Wise FW. Raman-scattering study of exciton-phonon coupling in PbS nanocrystals. *Physical Review B* 1997, **55**(15)**:** 9860-9865.

4. Ovsyannikov SV, Shchennikov VV, Cantarero A, Cros A, Titov AN. Raman spectra of $(PbS)_{1.18}(TiS_2)_2$ misfit compound. *Mater Sci Eng A* 2007, **462**(1–2)**:** 422-426.

5. Kang J-G, Lee G-H, Park K-S, Kim S-O, Lee S, Kim D-W*, et al.* Three-dimensional hierarchical self-supported multi-walled carbon nanotubes/tin(IV) disulfide nanosheets heterostructure electrodes for high power Li ion batteries. *Journal of Materials Chemistry* 2012, **22**(18)**:** 9330-9337.

6. Wang C, Tang K, Yang Q, Qian Y. Raman scattering, far infrared spectrum and photoluminescence of SnS2 nanocrystallites. *Chemical Physics Letters* 2002, **357**(5–6)**:** 371-375.

7. Smith AJ, Meek PE, Liang WY. Raman scattering studies of SnS_2 and $SnSe_2$. *J Phys C* 1977, **10**(8)**:** 1321-1333.

8. Smith GD, Firth S, Clark RJH, Cardona M. First- and second-order Raman spectra of galena (PbS). *Journal of Applied Physics* 2002, **92**(8)**:** 4375-4380.

9. Price LS, Parkin IP, Hardy AME, Clark RJH, Hibbert TG, Molloy KC. Atmospheric Pressure Chemical Vapor Deposition of Tin Sulfides (SnS, Sn_2S_3, and SnS_2) on Glass. *Chemistry of Materials* 1999, **11**(7)**:** 1792-1799.

10. Crampin S, Jensen H, Kröger J, Limot L, Berndt R. Resonator design for use in scanning tunneling spectroscopy studies of surface electron lifetimes. *Physical Review B* 2005, **72**(3)**:** 035443.

11. Maccariello D, Garnica M, Niño MA, Navío C, Perna P, Barja S*, et al.* Spatially Resolved, Site-Dependent Charge Transfer and Induced Magnetic Moment in TCNQ Adsorbed on Graphene. *Chemistry of Materials* 2014, **26**(9)**:** 2883-2890.

12. Wang S, Kuo K. Crystal lattices and crystal chemistry of cylindrite and franckeite. *Acta Crystallographica Section A: Foundations of Crystallography* 1991, **47**(4)**:** 381-392.

13. Williams TB, Hyde BG. Electron microscopy of cylindrite and franckeite. *Physics and Chemistry of Minerals* 1988, **15**(6)**:** 521-544.

14. Makovicky E, Petříček V, Dušek M, Topa D. The crystal structure of franckeite, Pb21. 7Sn9. 3Fe4. 0Sb8. 1S56. 9. *Am Mineral* 2011, **96**(11-12)**:** 1686-1702.

15. Shalvoy RB, Fisher GB, Stiles PJ. Bond ionicity and structural stability of some average-valence-five materials studied by x-ray photoemission. *Physical Review B* 1977, **15**(4)**:** 1680-1697.

16. Zhang YC, Qiao T, Hu XY, Wang GY, Wu X. Shape-controlled synthesis of PbS microcrystallites by mild solvothermal decomposition of a single-source molecular precursor. *Journal of crystal growth* 2005, **277**(1)**:** 518-523.

17. Cruz M, Morales J, Espinos JP, Sanz J. XRD, XPS and 119Sn NMR study of tin sulfides obtained by using chemical vapor transport methods. *Journal of Solid State Chemistry* 2003, **175**(2)**:** 359-365.

18. Zhang YC, Li J, Xu HY. One-step in situ solvothermal synthesis of SnS2/TiO2 nanocomposites with high performance in visible light-driven photocatalytic reduction of aqueous Cr(VI). *Applied Catalysis B: Environmental* 2012, **123–124:** 18-26.

19. Cheng S, Conibeer G. Physical properties of very thin SnS films deposited by thermal evaporation. *Thin Solid Films* 2011, **520**(2)**:** 837-841.

20. Zakaznova-Herzog VP, Harmer SL, Nesbitt HW, Bancroft GM, Flemming R, Pratt AR. High resolution XPS study of the large-band-gap semiconductor stibnite (Sb2S3): Structural contributions and surface reconstruction. *Surface Science* 2006, **600**(2)**:** 348-356.

21. Wang S, Kuo KH. Crystal lattices and crystal chemistry of cylindrite and franckeite. *Acta Crystallographica Section A* 1991, **A47**(4)**:** 381-392.

22. Makovicky E, Petříček V, Dušek M, Topa D. The crystal structure of franckeite, $Pb_{21.7}Sn_{9.3}Fe_{4.0}Sb_{8.1}S_{56.9}$. *American Mineralogist* 2011, **96**(11-12)**:** 1686-1702.

23. Mehner H. Mössbauer Investigations on Minerals of the Franckeite - Cylindrite Group. *Hyperfine Interactions* 1998, **112**(1)**:** 239-241.

24. Withers F, Yang H, Britnell L, Rooney AP, Lewis E, Felten A*, et al.* Heterostructures Produced from Nanosheet-Based Inks. *Nano Letters* 2014, **14**(7)**:** 3987-3992.

25. Evangeli C, Gillemot K, Leary E, González MT, Rubio-Bollinger G, Lambert CJ*, et al.* Engineering the Thermopower of C60 Molecular Junctions. *Nano Letters* 2013, **13**(5)**:** 2141-2145.

26. Rincon-Garcia L, Ismael AK, Evangeli C, Grace I, Rubio-Bollinger G, Porfyrakis K*, et al.* Molecular design and control of fullerene-based bi-thermoelectric materials. *Nat Mater* 2016, **15**(3)**:** 289-293.

27. Evangeli C, Matt M, Rincón-García L, Pauly F, Nielaba P, Rubio-Bollinger G*, et al.* Quantum Thermopower of Metallic Atomic-Size Contacts at Room Temperature. *Nano Letters* 2015, **15**(2)**:** 1006-1011.

28. Buscema M, Island JO, Groenendijk DJ, Blanter SI, Steele GA, van der Zant HS*, et al.* Photocurrent generation with two-dimensional van der Waals semiconductors. *Chemical Society Reviews* 2015, **44**(11)**:** 3691-3718.

29. Furchi MM, Polyushkin DK, Pospischil A, Mueller T. Mechanisms of Photoconductivity in Atomically Thin MoS2. *Nano Letters* 2014, **14**(11)**:** 6165-6170.

30. Ortiz-Conde A, García Sánchez FJ, Muci J. Exact analytical solutions of the forward non-ideal diode equation with series and shunt parasitic resistances. *Solid-State Electronics* 2000, **44**(10)**:** 1861-1864.

31. Choi MS, Qu D, Lee D, Liu X, Watanabe K, Taniguchi T*, et al.* Lateral MoS2 p–n Junction Formed by Chemical Doping for Use in High-Performance Optoelectronics. *ACS Nano* 2014, **8**(9)**:** 9332-9340.

32. Deng Y, Luo Z, Conrad NJ, Liu H, Gong Y, Najmaei S*, et al.* Black Phosphorus–Monolayer MoS2 van der Waals Heterojunction p–n Diode. *ACS Nano* 2014, **8**(8)**:** 8292-8299.

33. Furchi MM, Pospischil A, Libisch F, Burgdörfer J, Mueller T. Photovoltaic Effect in an Electrically Tunable van der Waals Heterojunction. *Nano Letters* 2014, **14**(8)**:** 4785-4791.
